# Time-series RNA metabarcoding of the active *Populus tremuloides* root microbiome reveals hidden temporal dynamics and dormant core members

**DOI:** 10.1128/msystems.00285-25

**Published:** 2025-11-07

**Authors:** Jake Nash, Keaton Tremble, Christopher Schadt, Melissa A. Cregger, Corbin Bryan, Rytas Vilgalys

**Affiliations:** 1 Department of Biology, Duke University 3065, Durham, North Carolina, USA; 2 Biosciences Division, Oak Ridge National Laboratory 6146, Oak Ridge, Tennessee, USA; University of Hawaiʻi at Mānoa, Honolulu, Hawaii, USA

**Keywords:** time-series, RNA metabarcoding, *Populus *root, microbiome

## Abstract

The rhizosphere is a critical interface between plant roots and soil, harboring diverse microbial communities that are essential to plant and ecosystem health. Although these communities exhibit stark temporal dynamics, their dormancy/activity transitions remain poorly understood. Such transitions may enable microbes to rapidly adjust functional contributions faster than community turnover alone would allow. Here, we used RNA metabarcoding to characterize the active fraction of microbial communities on the roots of quaking aspen (*Populus tremuloides*) in a time-series study across a natural environmental gradient. We explore cryptic temporal microbial community dynamics of rhizosphere communities at the ecosystem scale. The active rhizosphere bacterial and fungal communities were more temporally dynamic than total communities, while total communities exhibited a stronger response to site-specific conditions. Notably, some core microbiome members were often inactive, yielding a smaller “active core” subset. The fungal endophyte *Hyaloscypha finlandica* was the only microbe that was both present and active in all plots across all timepoints. Soil temperature strongly influenced both total and active community composition, with the fungal class Eurotiomycetes showing a temperature-dependent seasonal decline in abundance. Together, these results reveal that modulation of microbial activity levels is a key mechanism by which the plant root holobiont responds to environmental variation, and that even dominant symbionts may frequently persist in dormancy within the rhizosphere.

**IMPORTANCE:**

Members of the rhizosphere exhibit dynamic patterns of activity and dormancy. This study stresses the need to focus on active microbial communities to detect temporal changes in plant microbiomes. Additionally, the metabolic activity of microbes should be considered a key determinant of core microbiome membership. Parallel patterns in active community dynamics between fungal and bacterial communities provide a potentially generalizable rule of microbial community temporal dynamics in plant rhizospheres.

## INTRODUCTION

Plant roots are host to assemblages of endophytic, epiphytic, and rhizospheric microbes that can facilitate nutrient uptake, boost plant immunity, and increase resistance to abiotic stress (1, 2). Large-scale plant root and soil microbiome studies have found that both soil chemistry and climate have strong effects on belowground microbial community composition (3–5). In addition to the clear impact of soil and climate on microbial communities, individual extreme environmental events such as droughts, heat waves, and wildfires can cause rapid turnover in microbial community composition, likely due to variation in stress tolerance among community members (6, 7). Microbes can also transition between states of metabolic dormancy or activity in response to environmental cues. The existence of metabolically inactive microbes has led some microbial ecologists to divide microbial communities into a *total community*, comprised of the full complement of microbes present in an environment, and an *active community*, comprised of the subset of microbes that are metabolically active (8, 9). The ability of plant-symbiotic microbes to perform essential functions in plant root microbiomes, such as nutrient uptake and conferment of stress resistance, depends on the ability of microbes to remain metabolically active under variable environmental conditions (10, 11). Thus, it is essential to understand not only how environmental variation impacts total communities, but specifically how such variation affects the ecological dynamics of active communities. It has been shown that total microbial communities are structured by classic ecological processes, such as ecological filtering, biotic interactions, and dispersal limitation (12, 13). However, active community composition is likely also mediated by immediate environmental conditions that cause rapid transitions between dormancy and activity (8, 14). Still, we lack an understanding of the importance of short-term environmental fluctuations relative to longer-term climatic variation in structuring active microbial communities. This knowledge gap complicates predictions of the influence of microbial communities on ecosystem processes, which will be essential for the incorporation of microbial community data into ecosystem models.

Bacteria and fungi can rapidly transition between states of dormancy and activity depending on suitable environmental conditions (14–17). Dormant microbes are defined as cells exhibiting very low levels of metabolism and can either be resistant propagules, such as spores and sclerotia, or vegetative cells with reduced metabolic activity and possibly the accumulation of specific metabolites (8, 18, 19). Turnover of the composition of active microbial communities has the potential to occur more quickly than changes to the total community because it only requires spore germination or induction of metabolism rather than changes to population size through cell division or death (20). Rapid changes in the activity of bacterial and fungal symbionts can thus provide a means for microbial systems to quickly respond to acute environmental changes, thus facilitating the maintenance of essential metabolic processes (8, 9, 14, 21).

In addition to the taxa in plant microbiomes that fluctuate in response to the environment, most microbial systems also contain taxa that are shared among distinct communities and persistent through time, termed the “core microbiome” (22). Core microbiomes are thought to be highly important in supporting host fitness and may be selectively recruited by their hosts (23). The core microbiome is often quantitatively defined by microbes’ occupancy (the proportion of samples in which a microbe occurs), with occupancy thresholds (often 0.95 or above) used as a common criterion for inclusion in the “core.” Temporal stability in abundance is often also included as a criterion for core microbiome membership, thus requiring time-series sampling (22). The ability of core rhizosphere microbial taxa to deliver essential functions to plants is likely dependent not only on their ability to persist across variable environmental conditions but also to maintain metabolic activity under these environments and through time. However, few studies of core microbiomes have tested whether these taxa are consistently active through time, and thus it is possible that some core taxa are widely present as dormant propagules but have little functional importance for communities. Experimental assessments of the “active core microbiome” in the rhizosphere could thus further clarify which core microbiota are most important in supporting host acclimation to a wide variety of environmental conditions.

DNA metabarcoding is widely used to profile total microbial community composition through amplification of ribosomal marker genes. However, DNA metabarcoding can inflate the importance of dormant and dead microbes, which can constitute 40% or more of the taxa detected with DNA metabarcoding (8). In contrast to DNA-based studies, RNA metabarcoding is used to profile active microbial community composition and thus can better capture potentially important taxa for ecosystem and host function. RNA metabarcoding usually relies on the amplification of cDNA generated from transcribed ribosomal genes, with the 16S subunit serving as a common target for bacteria and the ITS region serving as a common target for fungi (24, 25). The ITS region is transcribed as part of a short-lived primary rRNA precursor containing the small subunit, 5.8S region, and large subunit (26). The highly transient nature of transcribed ITS regions means that they can serve as an indicator of cellular metabolic activity. However, because ribosomes can persist in resting spores and other inactive cells, 16S RNA metabarcoding may have less power to discriminate the active from the total community (27, 28), which subtly distinguishes the insights available from ITS metabarcoding of fungi and 16S metabarcoding of bacteria. Although dual DNA/RNA metabarcoding can be powerful to discriminate active from total communities, this approach is still impacted by known methodological limitations to metabarcoding, such as variable ribosomal copy number and difficulties with the interpretation of relative abundance data. Variation in ribosomal copy number is present in both genomes and functional ribosomes, which would impact DNA and RNA metabarcoding approaches, respectively. This variation is present both within and among species (29–31) and can potentially bias estimates of microbial diversity and community composition. Furthermore, unless specialized protocols are used, metabarcoding only provides information on relative abundances, which makes it difficult to determine whether the shift in a taxon’s abundance is due to fluctuations in abundances of other taxa in the community (32).

Trees in the genus *Populus* are important model species for understanding plant responses to abiotic stress and plant-microbe interaction (33–35). *Populus* spp. are dual-mycorrhizal, able to associate with both arbuscular mycorrhizal and ectomycorrhizal fungi, as well as the melanized dark septate endophytes that colonize roots without obvious symptoms (36–38). The surface (rhizoplane), interior (endosphere), and surrounding soil (rhizosphere) of *Populus* roots are also colonized by a diverse bacterial community dominated by Pseudomonadota, Actinomycetota, Bacteroidota, and Bacillota (36, 39, 40). These bacteria express a wide array of molecular functions, including production of antibiotics, secondary metabolites, plant hormones, and siderophores (41).

**Fig 1:**
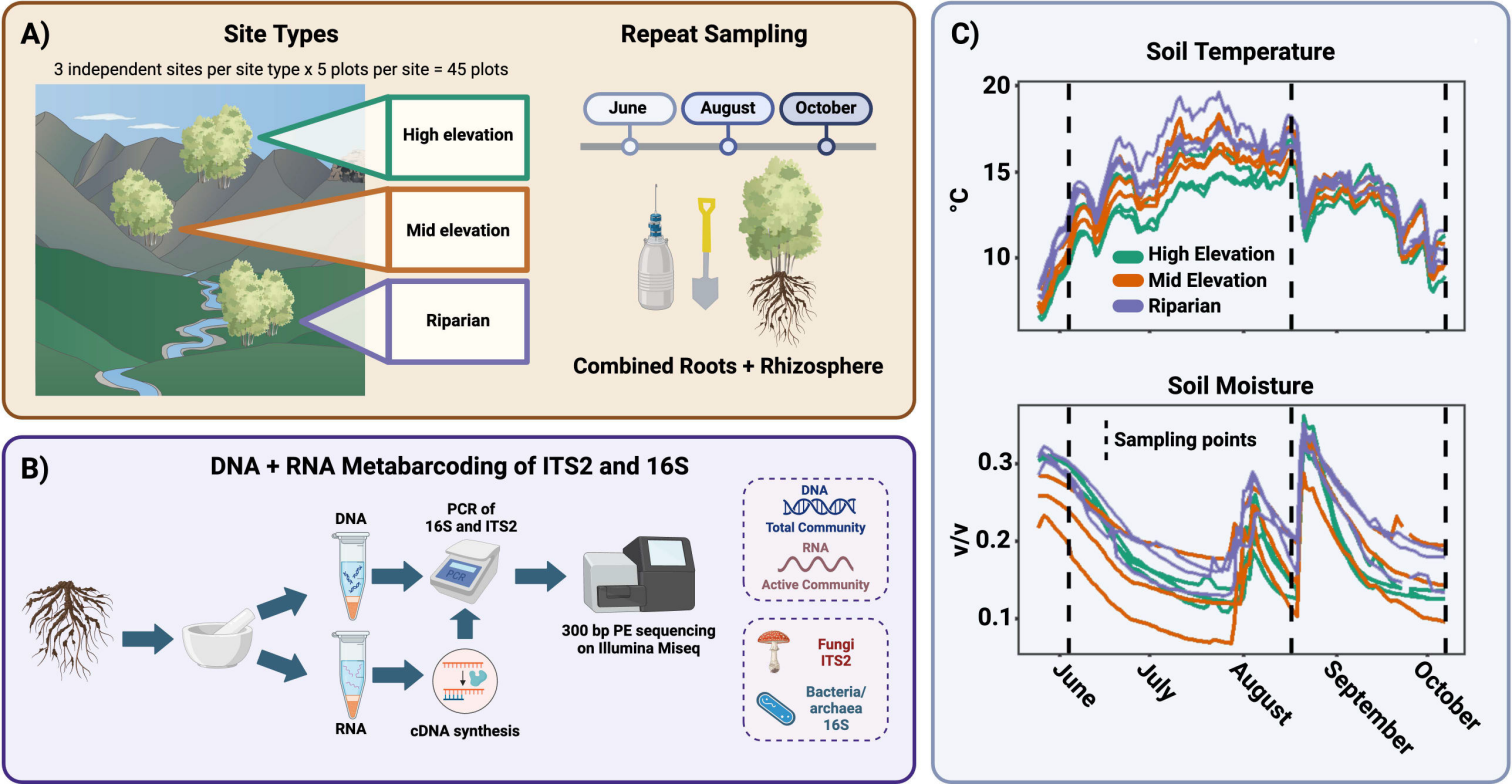
(**A**) The field sampling design is depicted. A mixed root/rhizosphere sample was collected from three site types, with three independently replicated sites per site type. Each of these nine sites had five plots collected along a transect, for a total of 45 plots. These 45 plots were sampled three times in June, August, and October for a total of 135 samples. (**B**) Samples were ground and subjected to parallel RNA/DNA metabarcoding for ITS2 (fungi) and 16S (bacteria/archaea) and sequenced on an Illumina MiSeq with 300 bp paired-end sequencing. (**C**) Average soil moisture and temperature for each site are measured with in-ground sensors collecting data every 15 minutes.

We utilized time-series sampling that leveraged the stark spatial and seasonal environmental variation in *P. tremuloides* stands in the Uinta Mountains in northeastern Utah to test the following hypotheses: (i) active microbial communities will be more temporally dynamic than total microbial communities, (ii) microbial taxa will exhibit unique responses to spatial and temporal environmental variation, and (iii) there will be a core microbiome comprised of highly metabolically active microbes across spatially variable sites that persists through time. In the Uinta Mountains, *P. tremuloides* occupies habitat types, including mesic riparian sites, mid-elevation semi-arid sites, and high-elevation montane sites, which we targeted in a hierarchical sampling design with stand-level ecological replication (see Fig. 1). These sites experience high levels of seasonal variation in moisture and temperature associated with predictable snow melt, drought, and monsoon periods that we leveraged with a time-series sampling design to explore the impacts of temporal environmental variation on active microbial communities.

## MATERIALS AND METHODS

### Site description and sampling

We selected nine *P. tremuloides* stands (referred to as “sites”) located within a 6 km stretch along the Provo River valley in the Uinta–Wasatch–Cache National Forest across three habitat types: riparian, mid elevation, and high elevation (see Fig. 1). Sites were chosen where *P. tremuloides* constituted >90% of the tree stems to avoid confounding effects of coexisting overstory tree species. We confirmed the ecological distinctness of sites by collecting data on understory vegetation (see Fig. S1), soil chemistry (see Fig. S2), growing season soil moisture and temperature using *in situ* sensors (see Fig. S3), and seasonal ion fluxes (see Fig. S4; File S1). Riparian sites were located along 1–2 m wide streams feeding the Provo River, with sampling plots located within 2 m of the stream. Riparian sites had high understory cover of graminoids and (at one riparian site) *Equisetum* (see Fig. S1), elevated soil iron content, and the highest growing season mean soil temperature (14.4°C; see Fig. 1; Fig. S3). Mid-elevation and high-elevation sites were both upland habitats located on northeast to northwest facing slopes. Mid-elevation sites were intermixed with shrubs, whereas high-elevation *P. tremuloides* sites were interspersed with tracts of coniferous forest (see Fig. S1). High-elevation sites had elevated cation exchange capacity (*P* < 0.05), slightly elevated soil organic carbon (n.s.; see Fig. S2), and the coldest soils (see Fig. 1; Fig. S3). Soil moisture was not significantly different between site types but varied widely among individual sites and pronounced seasonal dynamics associated with snowmelt and monsoon storms (see Fig. 1; Fig. S3). Three independent, spatially separated sites were selected for each of the three site types. At each site, an 80 m transect was established, with five 2 m circular plots established along the transect with 20 m spacing that were selected to include a cluster of aspen stems. Aspen fine roots were sampled from each plot at three time points in a single growing season—early June, mid-August, and early October of 2021. Fine roots and adhering soil were sampled by digging within the top 15 cm of soil at the base of trees within the plot until approximately 2 g of root and adhering soil had been sampled. Samples were immediately flash-frozen in liquid nitrogen in the field and kept at −80°C until processing.

### Molecular analyses and bioinformatics

Detailed molecular and bioinformatics are provided in File S1, but a brief summary is provided here. DNA and RNA were extracted from each fine root sample, including adhering rhizosphere soil. RNA was treated with DNase to remove DNA contamination and reverse transcribed to generate cDNA, which was then used in a PCR metabarcoding workflow along with the DNA to generate 16S (for bacteria and archaea) and ITS2 (for fungi) libraries with standard primers (36, 42, 43). Although the 16S primers target both Bacteria and Archaea, we refer to that data set as the bacterial data set in this manuscript because Archaea were in very low abundance. Libraries were sequenced on two separate runs of an Illumina MiSeq with 300 base pair paired-end sequencing with v3 chemistry at Duke University’s Center for Genomic and Computational Biology. Sequence data were processed with QIIME 2 v.2021.11 (44) and DADA2 (45) and assigned taxonomy with the UNITE v.8.3 99% database for Fungi (46) and the SILVA version 138.2 99% database for Bacteria/Archaea (47, 48).

### Statistics

16S and ITS data were rarefied to 13,407 and 17,182 sequences, respectively, to achieve an even sequencing depth while retaining as many samples as possible. This generated tables of bacterial and fungal relative abundance that were used for all quantitative analyses. Alpha diversity metrics were calculated using the *phyloseq* package (49). The *lme4* package (50) was used to test the effects of site type, season, and community type (active vs total) on Shannon diversity with a random effect of site. Dissimilarity matrices were generated by performing square root transformation followed by Wisconsin double standardization (first standardizing taxon abundance by its maximum read abundance, then standardizing by the total read abundance per sample) before calculating Bray-Curtis dissimilarity, and then used to test for the effects of site type, site, plot, season, and metabarcoding method on fungal and bacterial community composition using the adonis2() function in the *vegan* package (51) with 999 permutations. Beta-diversity patterns were visualized using non-metric multidimensional scaling (NMDS) conducted on the same dissimilarity matrices using the metaMDS() function with 100 iterations (51). The proportion of dormant taxa in each sample was calculated by dividing the number of active taxa (observed in the RNA data set) by the total number of taxa (observed in either the RNA or DNA data set) and then subtracting that ratio from 1. Random forest modeling was used to identify potentially important linkages between soil/environmental data and microbial community NMDS axes and abundances of dominant taxa (52). In cases where soil covariates were highly correlated, they were combined into a single index variable using principal component analysis before being used as a predictor in the random forest models. Notably, this resulted in cation exchange capacity, soil calcium content, and total organic carbon being collapsed into a single measure of soil organic matter, and pH and base saturation being collapsed into a single measure of soil acidity. Relationships identified by random forests were further validated using mixed models and visual inspection of data to ensure model robustness (50). For significant relationships, we compared mixed models using both a fixed slope as well as a site-dependent random slope. Models were then compared using a likelihood ratio, and significant differences in the log-likelihood ratio of the models were interpreted as indicators of scale dependence of the association (i.e., within-site slopes being distinct from between-site slopes). We did not test for scale dependence for the soil sensor-derived soil moisture and temperature data because the sensors were only installed at three of the five plots at each site, which prevented a statistically powerful estimation of site-specific slopes. The betadisp() function in *vegan* (51) was used to test for differences in beta dispersion across site types and seasons. LinDA (53) was used with a mixed model formula with site included as a random factor to identify differences in taxon abundance in response to community type (active vs. total), site type, and season. Mantel tests were used to test for distance decay of community composition and validated by doing partial Mantel tests that first accounted for effects of soil chemistry before testing for true distance effects (51).

## RESULTS

### Response of total and active communities to spatiotemporal environmental variation

**Fig 2:**
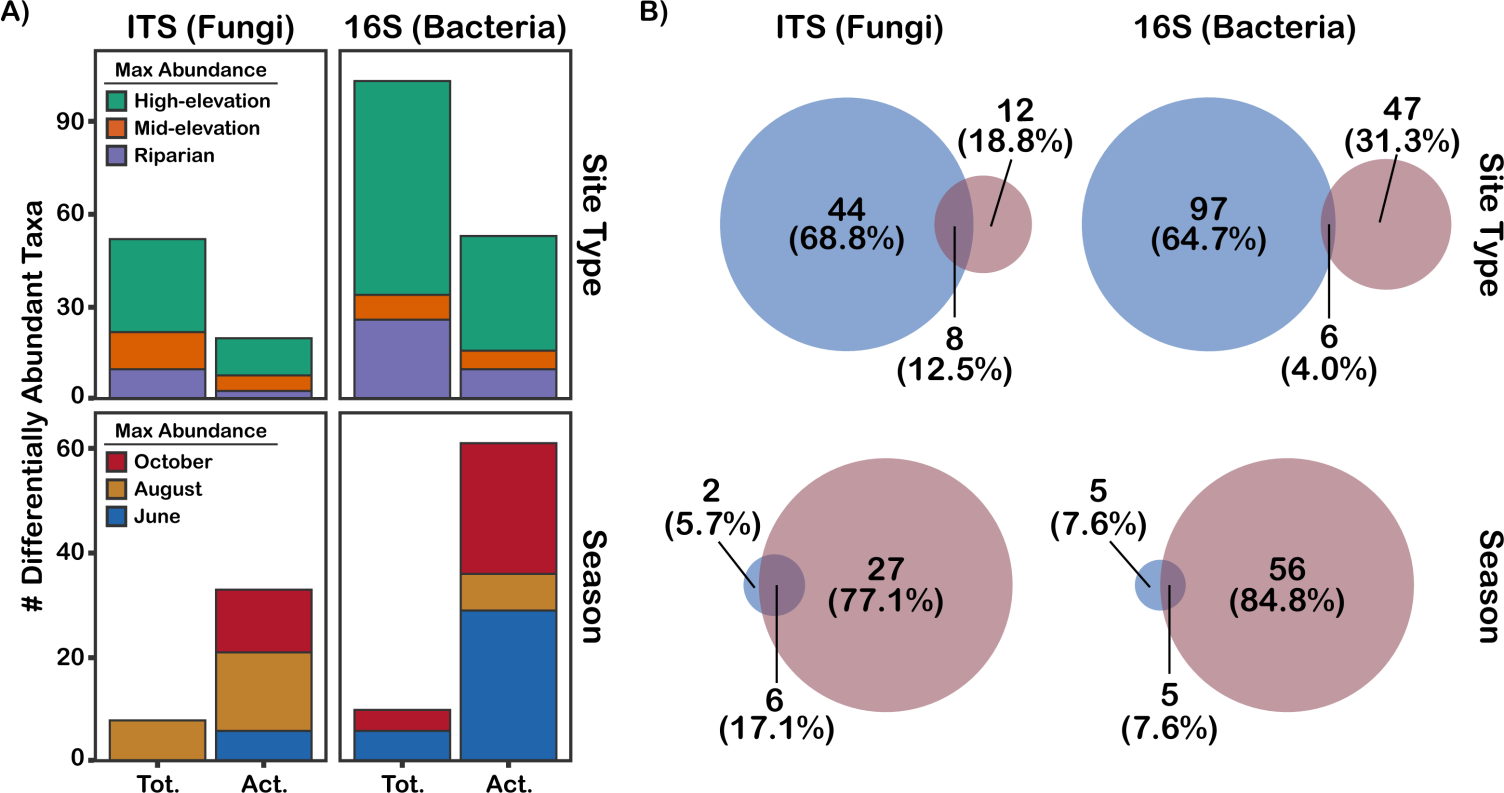
Results of LinDA differential abundance tests for differences in taxon abundance between site types and seasons. (**A**) The number of differentially abundant taxa detected in the total (tot.) and active (act.) communities, color-coded by the treatment (site type or season) where the taxa reached their maximum abundance. (**B**) Venn diagrams showing the taxa detected as differentially abundant across site types and seasons in active and total communities. The blue Venn circles represent the number of taxa detected as differentially abundant in the total community data set, and the red Venn circles represent the number of taxa detected as differentially abundant in the active community. The area of the Venn circles is proportional to the number of taxa.

**Fig 3:**
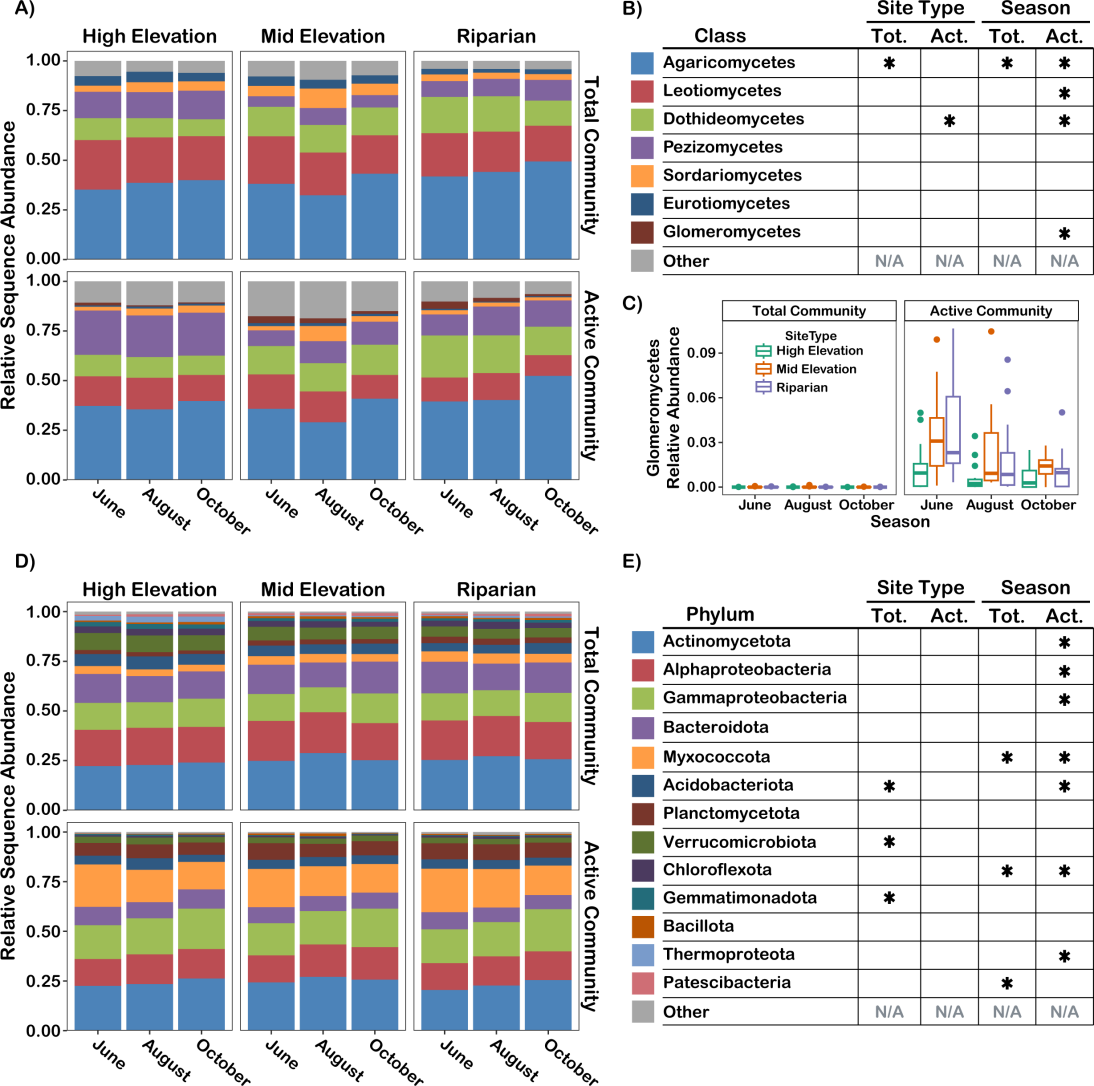
(**A**) Relative sequence abundance of dominant fungal classes (>0.1% relative sequence abundance) across site types and seasons in the active and total communities. (**B**) Legend and significance of site type and season effects on the dominant fungal classes in the total (tot.) and active (act.) communities determined by LinDA differential abundance tests. (**C**) Relative abundance of the arbuscular mycorrhizal Glomeromycetes across site types and seasons in the active and total communities. (**D**) Relative sequence abundance of dominant bacterial phyla (>0.1% relative sequence abundance) across site types and seasons in the active and total communities. Note that for Pseudomonadota, data were examined at the class level. (**E**) Legend and significance of site type and season effects on the dominant bacterial and archaeal phyla in the total (tot.) and active (act.) communities determined by LinDA differential abundance tests. For (**B**) and (**E**), * denotes significance at any level (*P*_adj_ < 0.05), and N/A indicates that statistical tests were not performed for low abundance lineages included in the “other” category.

Bacterial and fungal total communities were more strongly structured by site type than were active communities, while active communities had stronger seasonal dynamics than total communities. PERMANOVA found that community composition was more strongly structured by site type (ITS2: R^2^ = 0.067, *P* = 0.001; 16S: R^2^ = 0.061, *P* = 0.001) than by season (ITS2: R^2^ = 0.015, *P* = 0.001; 16S: R^2^ = 0.013, *P* = 0.001) for both fungal and bacterial communities (see Tables S4 and S5), although the effect of site type was stronger in the total community (ITS2: R^2^ = 0.11, *P* = 0.001; 16S: R^2^ = 0.11, *P* = 0.001) than in the active community (ITS2: R^2^ = 0.073, *P* = 0.001; 16S: R^2^ = 0.074, *P* = 0.001). Conversely, seasonality had a greater effect on community composition in the active community (ITS2: R^2^ = 0.026, *P* = 0.001; 16S: R^2^ = 0.024, *P* = 0.001) than in the total community (ITS2: R^2^ = 0.016, *P* = 0.001; 16S: R^2^ = 0.018, *P* = 0.003; see Tables S6 to S9). Follow-up tests to address the potential for confounding differences in beta-dispersion—beta-dispersion tests and W_d_*-tests (54)—confirmed that site type had a strong effect on fungal and bacterial active and total community composition, while effects of seasonality were only confirmed in the bacterial active community. We also quantified the degree of community responsiveness to site type and season in the total and active communities by identifying significantly differentially abundant taxa. In the total community, more taxa varied in abundance by site type than by season (52 vs. 8 fungal operational taxonomic units [OTUs]; 103 vs. 10 bacterial amplicon sequence variants [ASVs]), whereas in the active community, more taxa varied by season than by site type (33 vs. 20 fungal OTUs; 61 vs. 53 bacterial ASVs). There was little overlap in the assemblages of taxa found to be variable by site type or season between the active and total communities (see Fig. 2B). Most of the taxa that varied in abundance by site type reached peak abundance in the high-elevation sites in both the active and total communities, indicating a distinct community inhabiting high-elevation sites. Among the high-elevation specialists (for which both total community and active community tests were significant), there were three ectomycorrhizal fungi (*Hebeloma crustuliniforme*, *Hebeloma mesophaeum*, and *Geopora tolucana*), two potential dark septate endophytes in the Helotiales (*Varicosporium* sp. and *Tetracladium* sp.), and six bacteria (Amycolatopsis sp., Umezawaea sp., Ohtaekwangia sp., Haliangiaceae sp., Chitinophagaceae sp., and Cryptosporangium sp.). Higher fungal lineages had similar abundances across site types (see Fig. 3A and B), whereas the bacterial lineages Acidobacteriota, Verrucomicrobiota, and Gemmatimonadota were in greater abundance at high-elevation sites in the total community (see Fig. 3D and E). Many higher fungal and bacterial lineages displayed seasonal fluctuations in abundance in the active community, but not in the total community (see Fig. 3).

**Fig 4:**
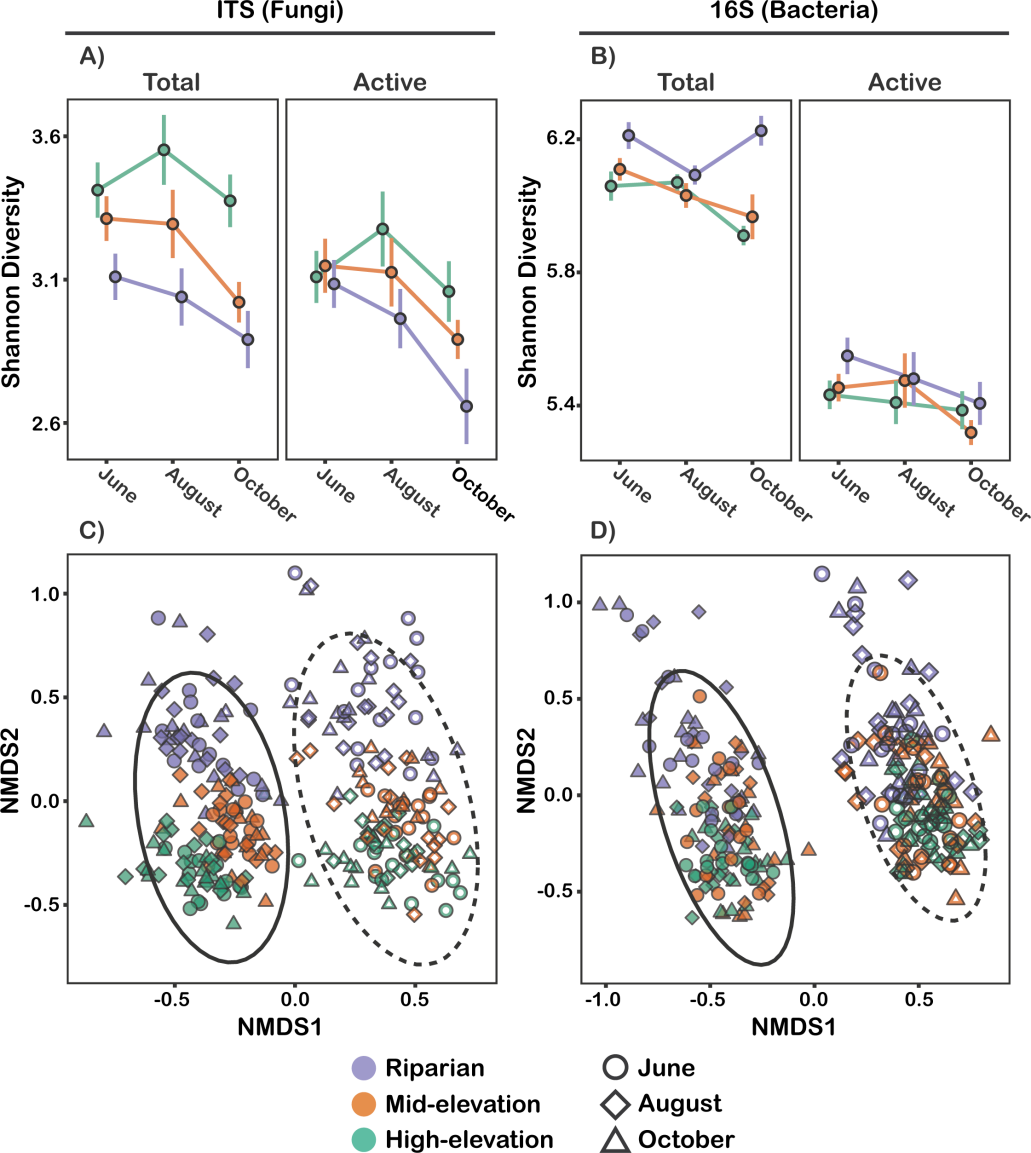
Alpha and beta diversity patterns for 16S and ITS structured by site type, season, and community type (active vs total). (**A**) Fungal communities displayed a general decrease in diversity throughout the season, whereas bacterial communities did not (**B**). Active communities were less diverse than total communities. (**C, D**) NMDS of fungal and bacterial community composition shows a strong differential between active (open points, dashed ellipse) and total (closed points, solid ellipse) communities. Site type had the greatest effect on community composition, and season had a lesser effect.

Within-site beta dispersion was greatest at riparian sites and lowest at high-elevation sites and tended to increase throughout the growing season for both fungal and bacterial communities (see Fig. S5). Additionally, within-site beta dispersion of fungal and bacterial communities was greater in the active community than in the total community (see Fig. S5; Tables S10 and S11), indicating greater patchiness in the distribution of active taxa. Metabarcoding of active and total communities both found a general decrease in fungal (*P* < 0.0001; Table S2) and bacterial (*P* = 0.003; Table S3) Shannon diversity throughout the growing season, with some site type-specific exceptions (see Fig. 4A and B). Fungal Shannon diversity was the highest (*P* < 0.0001) and most seasonally stable at the high-elevation sites, while bacterial Shannon diversity peaked at the Riparian sites (*P* < 0.0001). We found strong evidence for spatial structure in community composition. Site and plot both had strong effects on fungal and bacterial composition, with plot effects (R^2^ = 0.25–0.29) being stronger than both site (R^2^ = 0.11–0.14) and site type effects (R^2^ = 0.07–0.11). This pattern demonstrates that fine-scale differences in community composition between microsites within the same stand were maintained across seasons, despite some seasonal turnover. There were significant distance decay relationships in the total and active fungal and bacterial communities at all timepoints across the range of distances from 15 m to >8 km (r = 0.41–0.57, *P* < 0.001; see Fig. S6). Distance decay patterns were still strongly significant and highly predictive when variation in soil nutrients was accounted for (r = 0.38–0.56, *P* < 0.001), suggesting that spatial structuring of community composition was primarily driven by dispersal limitation rather than by nutrients, although nutrients still had a distinct effect on community structure (described below).

**Fig 5:**
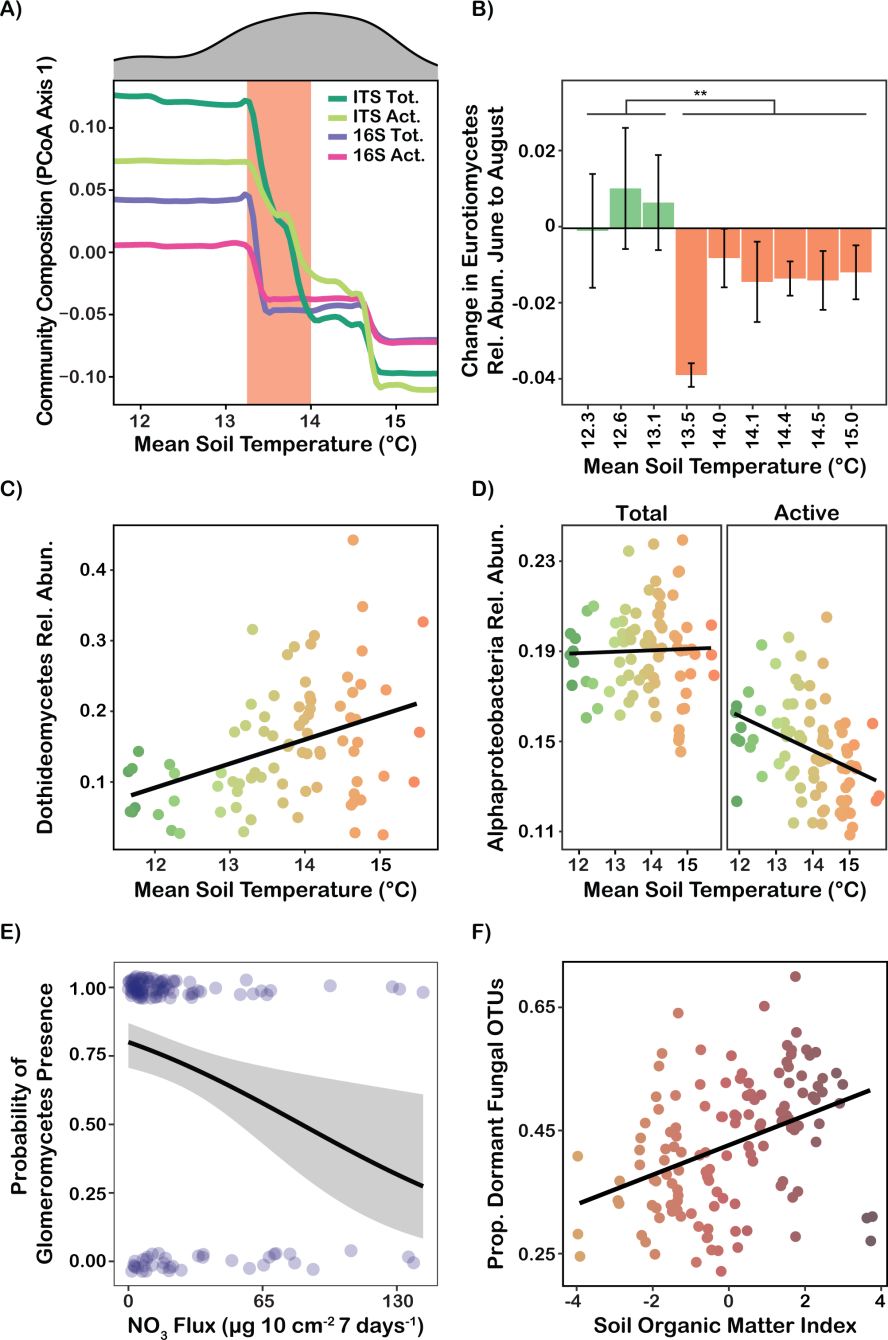
Linkages between microbial community properties and environmental soil factors. (**A**) A partial dependence plot from random forest models showing the response of NMDS axis 1 from the four datasets (ITS total community, ITS active community, 16S total community, and 16S active community) against mean soil temperature. (**B**) Differences in Eurotiomycetes relative abundance in the total community between the June and August timepoints, with averages and standard errors displayed for each site. Negative values indicate a decline in abundance from June to August. ** indicates *P* < 0.01. (**C**) The relationship between Dothideomycetes relative abundance and mean soil temperature in the active community. Point color corresponds to the mean soil temperature. (**D**) The relationship between Alphaproteobacteria relative abundance and mean soil temperature in the total and active communities. Point color corresponds to the mean soil temperature. (**E**) A glm model based on a logistic distribution to predict the probability of Glomeromycetes presence as a function of nitrate flux. Points at 1 indicate samples where Glomeromycetes were present, and those at 0 indicate samples where Glomeromycetes were absent. (**F**) The proportion of dormant fungal OTUs as a function of an index of soil organic matter content (combining total organic carbon, cation exchange capacity, and soil calcium). Points are colored by this index.

Multivariate analyses identified mean soil temperature, soil organic matter, pH, soil iron content, base saturation, and grass cover as strong predictors of active and total fungal and bacterial community composition (random forest IncMSE: 10%–25%; see File S4). Community composition of active and total fungal and bacterial communities changed rapidly in response to mean soil temperature between 13.3°C and 14°C, but was relatively stable outside of this range, suggesting a soil temperature threshold that differentiated high- and low-temperature-adapted microbial communities (see Fig. 5A). At the six sites with the highest soil temperature (≥13.5°C), Eurotiomycetes exhibited a decline in abundance from the June to August time point in the total community, while at the three sites with the lowest soil temperature, Eurotiomycetes abundance remained stable during this time period (*P* = 0.004; see Fig. 5B). Eurotiomycetes abundance did not recover between the August and October time points to the levels observed at the June timepoint. The August time point came shortly after a period of the warmest soil temperatures, suggesting that this soil temperature threshold acted as a seasonally dependent environmental filter on Eurotiomycetes abundance. Dothideomycetes had a consistently positive response to soil temperature with no evidence of a threshold effect or a seasonal interaction (DNA and RNA: *P* = 0.0027; see Fig. 5C). The positive response of Dothideomycetes to soil temperature was largely driven by a single OTU identified as *Aquilomyces patris*, which is a dark septate endophyte that was the third most abundant fungal OTU in the data set. Alphaproteobacteria activity was negatively associated with soil temperature (*P* = 0.022), while there was no correlation between Alphaproteobacteria abundance and soil temperature (*P* = 0.82; see Fig. 5D). Nitrate flux was a strong negative control on Glomeromycetes colonization (RNA: *P* = 0.0047; see Fig. 5E), but had a positive association with Thermoproteota activity and abundance (DNA and RNA: *P* < 0.001; see Fig. S7). All of the Thermoproteota ASVs in this data set were in the family *Nitrososphaeraceae,* which is a group of ammonia-oxidizing Archaea suggesting a strong role for this group in mediating rhizosphere nitrogen cycling. Thermoproteota also had a positive association with soil organic matter (Thermoproteota DNA: *P* = 0.046), while Alphaproteobacteria and Planctomycetota had negative associations with soil organic matter (Alphaproteobacteria DNA and RNA: *P* = 0.0039; Planctomycetota DNA and RNA: *P* = 0.033). We found that in most cases, environmental linkages were not scale-dependent, with consistent directionality and strength of associations when modeled as within or among site relationships. One notable exception to this was for the linkage between Thermoproteota and soil organic matter, which did exhibit scale dependence—the positive association emerged from the relationship between soil organic matter and Thermoproteota observed among sites, although there was not a strong relationship within sites. The proportion of fungi in dormancy was positively associated with soil organic matter content (*P* = 0.0029) and was consequently greater at the high elevation sites, which had more organic-matter-rich soils (*P* = 0.001). The proportion of bacteria in dormancy showed no significant relations with any soil or environmental data.

### Community differences between active and total communities

We identified 95 fungal taxa and 543 bacterial taxa that differed in abundance by at least 2× between the active and total communities (see File S4 for list). This represents 24% of fungal OTUs and 39% of bacterial ASVs that satisfied the filtering requirements of the LinDA tests used for differential abundance testing. Fungi in the arbuscular mycorrhizal class Glomeromycetes were dramatically overrepresented in the active community (1.84% sequence abundance) compared to the total community (0.0063% sequence abundance; *P* < 0.0001, see Fig. 3C). The bacterial phyla Patescibacteria and Thermoproteota were strongly enriched in the total community, while the Myxococcota and Planctomycetota were strongly enriched in the active community (see Fig. 3). Community composition was strongly differentiated between the active and total communities for both fungi (R^2^ = 0.06, *P* < 0.001; see Fig. 4C; Table S4) and bacteria (R^2^ = 0.12, *P* < 0.001; see Fig. 4D; Table S5). Taxon richness was significantly greater in the total community than in the active community for fungi (193 vs. 139 OTUs/sample, *P* < 0.0001) and bacteria (746 vs 451 ASVs/sample, *P* < 0.0001). On average, 47% (±9%) of fungal OTUs and 34% (±6%) of bacterial ASVs that occurred in a given total community sample were detected in the corresponding active community sample, suggesting that the active community represented a subset of the total community.

### Identification of an active core microbiome

In addition to identifying microbes within the *Populus* root microbiome that varied by site type and season, we also identified a core *Populus* root microbiome consisting of taxa that were present in at least 95% of samples across site types and seasons. We differentiated between a *total core microbiome* based on the DNA data set that contained 8 fungal and 50 bacterial taxa and an *active core microbiome* based on the RNA data set that contained only 2 fungal and 20 bacterial taxa (see Fig. S8; File S4 for list of taxa). Both total and active fungal core microbiomes were entirely comprised of Ascomycete OTUs. Although six out of the top ten most abundant fungal OTUs were ectomycorrhizal, no ectomycorrhizal fungi met the criteria for either total or active core microbiome membership because they occurred sporadically across samples. The bacterial active and total core microbiomes were primarily composed of Pseudomonadota and Actinomycetota, but also contained some members of the Acidobacteriota, Bacillota, Bacteroidota, Chloroflexota, Myxococcota, and Planctomycetota. The active core microbiome was generally a subset of the taxa in the total core microbiome (though there were two active core bacterial taxa that were not part of the total core community). Taxa identified as core in the total community were, in some cases, inactive in many of the samples in which they occurred (e.g., the fungus *Exophiala equina* and the bacteria *Mycobacterium* sp., *Acidiferrimicrobium* sp., *Phyllobacterium* sp., KD4-96 sp., Vicinamibacterales sp., and two *Acidibacter* spp.), and in one extreme case (the bacterium *Microlunatus* sp.) was found to be inactive in all samples where it was found.

## DISCUSSION

The rhizosphere is a critical interface between plant roots and the soil environment, harboring microbial communities that assist in nutrient acquisition and defense against pathogens. Although the structure of rhizosphere microbial communities is known to respond to shifting environmental conditions, it is unclear whether communities exhibit pronounced dynamics of metabolic activity and dormancy over time and through space. We hypothesized that (i) active fungal and bacterial communities would be more dynamic through time than total communities, (ii) microbial taxa would vary in abundance across ecologically distinct sites and between seasons, and (iii) core microbiome members would be highly metabolically active through space and time. Our results largely supported hypotheses i and ii—seasonal dynamics were more prevalent in the active community than in the total community, and there was a strong imprint of spatiotemporal variability on both active and total communities. In contrast, hypothesis iii was only partially supported—while many core taxa were consistently active, there was a sizable fraction of the core microbiome that was frequently inactive.

Our field study revealed distinct impacts of spatiotemporal environmental variation on the *Populus* root microbiome and its activity patterns. Environmental variation associated with site type had a greater impact on *total microbial community* composition than did temporal variation associated with seasonal changes. Conversely, temporal variation in environmental properties associated with seasonal changes had a strong impact on a large number of fungal and bacterial taxa in the *active community*. There were many fungi and bacteria that exhibited changes in abundance by season in the active community, but not in the total community, suggesting that these changes were due to modulation of metabolic rates rather than fluctuations in population size. Modulation of metabolic activity levels may enable the persistence of taxa during unfavorable periods, thus buffering microbial communities against environmental change (8).

Variation in rhizosphere microbial community structure was likely driven by soil temperature, organic matter content, pH, iron, and understory vegetation composition, consistent with a large body of work demonstrating the importance of these factors in structuring belowground microbial communities (3, 5, 55–61). These relationships were generally not scale-dependent, exhibiting similar qualitative patterns when examined either within or among sites. However, we did find that Thermoproteota exhibited a scale-dependent positive response to soil organic matter driven by among-site variation, despite the lack of a within-site relationship. Fungal and bacterial communities were subject to a strong soil temperature threshold effect that resulted in rapid community changes between 13.3°C and 14°C, but relative stability outside of this range. This finding implies that rhizosphere communities that exist at the precipice of this threshold may be highly vulnerable to further warming, and it is unclear whether transitions in community composition across this threshold would be readily reversible. However, this threshold should be interpreted within the local scale of our field experiment and should not be extrapolated to microbial communities in other climatic zones that are likely preadapted to distinct historical temperatures (62, 63). Within the single season of sampling that we conducted, we observed declines in Eurotiomycetes abundance during the hottest period of the year only at the sites that were warmer than this soil temperature threshold. These findings suggest that temperature-driven changes to rhizosphere microbial community composition may primarily be driven by short-term environmental extremes, although slower “press” disturbances also have well-documented effects on belowground microbial communities (64). We found that Alphaproteobacteria activity declined with increasing soil temperature without any corresponding change in Alphaproteobacteria abundance. This demonstrates that even if microbial taxa are able to persist in the face of environmental changes, their metabolic activity may be reduced, thereby preventing them from fulfilling their metabolic functional roles. Some microbial community members benefited from warmer soil temperatures. The dark septate fungal endophyte *Aquilomyces patris* was the third most abundant OTU in our data set and demonstrated a positive temperature response. Based on a search of the GlobalFungi database, *A. patris* is a common root symbiont of *Populus* and occurs across the semi-arid western United States (65), and it was originally isolated from *P. tremula* growing in a semi-arid habitat in Hungary (66). These observations suggest a possible role of *A. patris* as a stress-adapted fungal endophyte. Variation in soil nutrient status was also found to be an important determinant of community structure. Nitrate flux was negatively associated with the probability of Glomeromycetes colonization in the active community. Fungi in the Glomeromycetes are responsible for the formation of arbuscular mycorrhizae, which have been demonstrated through many fertilization experiments to be highly sensitive to inorganic nitrogen levels (67–69). The ammonia-oxidizing archaeal family *Nitrososphaeraceae* (phylum Thermoproteota) was positively associated with nitrate flux. Available ammonia was present at low levels, suggesting its efficient conversion to nitrate. Members of *Nitrososphaeraceae* are well established as key ammonia oxidizers in both terrestrial and marine systems (70). The association between their abundance and nitrate flux in our study suggests that this group plays an important role in sustaining the rapid conversion of ammonia to nitrate in the *Populus* rhizosphere, thereby maintaining the supply of inorganic nitrogen to plant roots and their associated mycorrhizal and endophytic fungal symbionts.

In addition to the strong ecological imprint of site type on microbial community composition, we found even stronger effects of spatial factors on community composition. Spatial effects were detected at multiple levels, with a significant effect of site and an even more pronounced effect of plot on both fungal and bacterial community composition. In light of our sampling design with a 2 meter plot size located along transects within stands, this suggests that microsites within forests separated by just tens of meters contain distinct assemblages of rhizosphere microbes that are maintained through time. Modeling of the distance decay relationship found that from a scale of 15 m to >8 km, rhizosphere microbial community composition became more dissimilar with increasing distance between plots, and that this relationship was better explained by spatial separation than by soil chemistry, suggesting dispersal limitation rather than soil environmental filtering as the dominant mechanism generating this pattern. Distance decay relationships have been frequently observed in many microbial systems and are likely a general feature of microbial communities, although the shape of the relationship can vary widely (12, 71). Additionally, we found an increase in within-site community dissimilarity (i.e., beta dispersion) for fungi and bacteria throughout the season. The increases in community dissimilarity and decreases in alpha diversity in the rhizosphere fungal community that we found may be signatures of host stress and dysbiosis due to the loss of a host’s ability to regulate its microbiota and successfully recruit mutualists (72, 73). Although it is unclear whether *P. tremuloides* trees in our study experienced increased stress throughout the growing season, our data provide tentative evidence for the breakdown of deterministic assembly later in the growing season, which may be due to a loss of host regulatory effects on the root microbiome or alternatively by a direct impact of stress on the rhizosphere microbiota.

Although site type was a strong determinant of community composition at the OTU/ASV level, higher-level bacterial and fungal lineages responded more strongly in their activity to season than to site type. Rhizosphere communities in the early spring displayed a greater activity of the fungal lineages Leotiomycetes and Glomeromycetes (containing arbuscular mycorrhizal fungi) and the bacterial lineage Myxococcota, but transitioned to increasing activity of the fungal lineage Agaricomycetes and bacterial lineages Actinomycetota, Alphaproteobacteria, and Gammaproteobacteria as the growing season progressed. The Leotiomycetes contain a number of fungal endophytes, and the Glomeromycetes are a monophyletic grouping of arbuscular mycorrhizal fungi. Both of these fungal groups are thought to have a lower carbon demand than ectomycorrhizal fungi (74, 75), which may explain their higher activity levels in early spring when trees may be carbon-limited due to recent leaf-out (76). Actinomycetota exhibited a modest increase in activity in October when soil moisture was the lowest and have been previously found to be enriched in plant root microbiomes subjected to experimentally induced drought conditions (77, 78). Actinomycetota have been hypothesized to have drought tolerance due to the production of quiescent resting spores (18, 79). However, our data provide evidence that Actinomycetota can maintain metabolic activity during periods of drought, suggesting the use of alternative mechanisms of drought tolerance than spore production. It is still unclear whether the seasonal changes in rhizosphere microbiome composition and diversity that we observed are indicative of a regular seasonal cycle due to our sampling within a single growing season. Another time-series study of *Populus* over multiple growing seasons found a continuous directional change in microbiome composition and limited cyclical seasonal patterns (80). *P. tremuloides* is a long-lived perennial with genets that can survive for centuries to millennia due to its clonal nature (81), and multi-year data sets will be particularly informative for resolving the temporal scales over which their root microbial communities develop. Multi-year monitoring of microbial communities would demonstrate whether rhizosphere microbial communities exhibit resilience to recover to a stable baseline state following seasonal changes and whether intra-annual community turnover is indicative of long-term trends (82).

Additionally, two of the lineages that varied in activity across seasons (Glomeromycetes and Myxococcota) were strongly enriched in the active community relative to the total community. Myxococcota have been previously documented as major components of the *Populus* rhizosphere and root endosphere (36) and have been found to be highly active in the uptake of plant carbon during root colonization in rice (83). Glomeromycetes are often scarcely represented in DNA-based amplicon surveys of plant roots and soils, but our study—along with two previous RNA metabarcoding studies (38, 84)—suggests this plant-mutualistic group is highly metabolically active and temporally dynamic. The much greater recovery of Glomeromycetes sequences in the RNA data set makes RNA metabarcoding a promising alternative to DNA metabarcoding to profile Glomeromycete communities without having to use taxon-specific primers (38). We did not find evidence that rare taxa were disproportionately active, as was found in a landmark study that compared total and active microbial communities in lake ecosystems (9).

In addition to the community members that varied across site types and seasons, we identified a core microbiome that was nearly universally present across sampling locations and through time. Based on the presence in the total community, this core consisted of 8 fungal and 50 bacterial taxa. However, we found that many core microbiome members were frequently inactive, with only 2 fungal and 20 bacterial taxa being included in the active core microbiome. Although many ectomycorrhizal fungi were highly abundant, they generally exhibited a patchy occurrence pattern and failed to meet the criteria for core microbiome membership in either the total or active community, which is consistent with another time-series study of *Populus* (33). The two active core fungal species were the dark septate endophyte *Hyaloscypha finlandica* and an unidentified Ascomycota sp. that had high sequence similarity to environmental sequences from plant roots and rhizosphere soil. *H. finlandica* has previously been demonstrated to be prevalent and highly metabolically active on potted *Populus* plants, suggesting a broad role as a core member of the *Populus* rhizosphere microbiome (38). Interestingly, we found that a core bacterial ASV identified as a *Mycobacterium* sp. was only active in 17% of samples. Although the *Mycobacterium* strain detected in our study is not closely related to human-derived isolates, another member of the genus that is implicated in Tuberculosis displays a pronounced dormant phase as part of the infection process (85), and the body-inhabiting strain *M. smegmatis* can also enter a dormant phase with low rates of RNA synthesis (86). These findings challenge the understanding of core microbiomes as essential and functionally important members of the microbiome (22). Instead, we find that some members have high capacities for dormancy and might only be classified as core members of the microbiome in DNA metabarcoding studies because of their persistence as dormant propagules or even as relic DNA from dead cells. Various criteria have been considered for core microbiome membership, including presence/absence, abundance, and network connectivity (22). Our study raises the question of whether persistent metabolic activity across a wide array of environmental conditions and through time should also be considered as a criterion for core microbiome membership.

Although our methodology relied on using ITS2 and 16S RNA metabarcoding as a measure of active microbes, it is possible that even RNA amplicons originated from dormant or dead cells. Ribosomes can persist in spores and other dormant cells that exhibit low rates of metabolic activity, which could have contributed to the 16S RNA metabarcoding community that we sampled. However, the ITS2 RNA metabarcoding method that we used to profile active fungal community composition likely originated from active, or very recently active, fungal cells because ITS2 is a short-lived component of ribosomal RNA that is promptly removed during ribosomal maturation. Despite this potential methodological caveat, it is possible that ribosomal content, even in dormant cells, is a useful indicator of *potential* metabolic activity, as indicated in a study that found that dormant persister cells of *Escherichia coli* with higher ribosomal number had faster rates of resuscitation following the cessation of antibiotics (87). Additional experimental approaches, such as probing active cells with deuterium-labeled water (88) or using metatranscriptomics to quantify cellular mRNA levels (89), would provide further confirmation of the dormancy patterns that we observed in this study and further validate whether cellular ribosomal content predicts cellular metabolic rates.

Our study has strong implications for ecosystem models of carbon and nutrient pools and fluxes. Although ecosystem models are increasingly including microbial community parameters (90), few models have incorporated microbial dormancy dynamics (14, 21, 91). Models that have incorporated dormancy have found higher projected carbon stocks (91) and greater stability in microbial biomass due to the quicker recovery that is enabled by the existence of a microbial seed bank (8, 9, 21). Additionally, modeling approaches have found that rates of dormancy are under strong environmental control and generally show a negative relationship with nutrient or carbon availability (8, 21). Conversely, our study found that higher rates of organic matter were *positively associated* with the fraction of dormant fungal OTUs, which counterintuitively implies that increasing substrate availability would induce dormancy. In cell cultures, carbon starvation is a well-documented trigger for microbial dormancy (19), and it is thus perplexing why increased soil organic carbon availability would be associated with *higher* levels of fungal dormancy. This finding certainly requires further validation in other ecosystems but would have profound implications for ecosystem models, possibly generating a feedback loop between organic matter content and microbial dormancy rates that could serve as a mechanism of soil organic carbon stabilization. Furthermore, our identification of an *active* core microbiome provides a means of prioritizing microbes that are most likely to contribute to the stability of global ecosystem processes. Models that tie community composition to changes to global nutrient fluxes might focus on changes to the metabolically active members of the core microbiome as a strong indicator of relevant microbial functional changes. Metagenomics and metatranscriptomics would provide strong validation that active core microbiota are important contributors to ecosystem processes (89, 92). We can hypothesize that these active core members perform molecular functions that are unique within the community, and thus the loss of these members (or their entry into dormancy) would be predicted to have major implications for the ability of communities to process nutrients.

To conclude, our study highlights a previously unrecognized layer of temporal dynamism within plant microbiomes, driven by the fluctuating metabolic activity of microbial symbionts. This dynamism likely contributes to the plasticity of genomically encoded, community-aggregated traits (92), which may offer adaptive advantages to plant hosts in a rapidly changing environment and also influence ecosystem processes like nutrient cycling and decomposition. This study makes a strong case for time-series molecular studies that focus on active microbial communities to detect community changes and provides a foundation for future research aimed at identifying the environmental stimuli and molecular processes that trigger transitions between microbial dormancy and activity. Additionally, we demonstrate that core microbiota are often inactive, and activity levels should be considered as a key criterion for including taxa in the core microbiome.

## Data Availability

The 16S and ITS reference sequences and raw sequence data generated during the current study have been submitted to GenBank and the Sequence Read Archive under the BioProject PRJNA1224426. Code underlying the analyses and figures presented here is available at https://github.com/jakenash12/PopulusActiveComm.

## SUPPLEMENTAL MATERIAL

File S1 msystems.00285-25-s0001.docx Supplemental methods.

File S2 msystems.00285-25-s0002.docx Figures S1 to S8.

File S3 msystems.00285-25-s0003.docx Tables S1 to S11.

File S4 msystems.00285-25-s0004.xlsb Supplemental resources and output of analyses.

ASM does not own the copyrights to Supplemental Material that may be linked to, or accessed through, an article. The authors have granted ASM a non-exclusive, world-wide license to publish the Supplemental Material files. Please contact the corresponding author directly for reuse.
