# Supplementary material for: Time-series RNA metabarcoding of the active Populus tremuloides root microbiome reveals hidden temporal dynamics and dormant core members
Source: mSystems. 2025 Nov 7;10(12):e00285-25. doi: 10.1128/msystems.00285-25 (PMC12710371; doi:10.1128/msystems.00285-25)
Supplement: File S1 — Supplemental methods. [file msystems.00285-25-s0001.docx]

**Methods**

*Soil and vegetation metadata collection*

Soil samples were taken from the top 15 cm of soil from each plot in May 2021 and were sent to the University of Georgia soil testing lab (Athens, GA) for soil chemistry analysis (see Fig. S2). We conducted understory vegetation surveys at each plot to measure the percent cover of grasses, forbs, shrubs, ferns, and bare ground using a radial point intercept method. The circular plots were divided into four quadrants and 12 point intercepts were scored in each quadrant (for 48 total intercepts) by dropping a pin flag randomly and recording the vegetation type of the first plant that it contacted. Ten days prior to each sampling date, four pairs of cation and anion ion exchange resin strips (Plant Root Simulators®, Western Ag, Saskatoon, Saskatchewan, Canada) were installed to provide an integrated measure of nutrient availability in the days leading up to sampling. Resin strips were left in the field for these ten days, removed when roots were sampled, and analyzed by Western Ag following their standard procedures. Teros 11 volumetric soil moisture and temperature sensors (Meter Group, Pullman, WA, USA) were installed at 15 cm depth in the three middle plots at each site to collect data at 15 minute intervals from late May until the final sampling point in October.

Although we had a largely intact time series of soil temperature and moisture data from the sensors, there were windows when individual sensors went offline due to equipment damage. For these windows of missing data, we interpolated data by developing linear models based on the closest online sensors’ data regressed against the sensor of interests’ data and then predicted the missing values based on these linear models. For both soil temperature and moisture, we calculated the following summary statistics – mean average change, minimum mean daily value, maximum mean daily value, average mean daily value, mean diel fluctuation, and range in mean daily value.

*Metabarcoding of plant roots*

The entirety of each fine root sample, including adhering rhizosphere soil, was cryogenically ground in a mortar and pestle with liquid nitrogen. DNA was extracted from 200 mg of ground roots using the DNeasy Powersoil Pro Kit (Qiagen, Hilden, Germany) and RNA from 50 mg of ground roots using the Spectrum™ Plant Total RNA Kit (Sigma-Aldrich, St Louis, MO, USA). RNA was reverse transcribed into cDNA using the Omniscript RT kit (Qiagen) with the primers LR5 (1), ITS4NGR (2, 3), and 806R (4). Amplicon libraries of 16S and ITS2 libraries were generated from DNA and cDNA using a two-step PCR protocol (see supplemental file S4). Amplicons were cleaned using the Omega Mag-Bind TotalPure NGS kit (Omega, Norcross, GA, USA) and pooled in equimolar quantities. ITS2 and 16S Libraries were sequenced on two separate runs of an Illumina MiSeq with 300 base pair paired end sequencing with v3 chemistry at Duke University’s Center for Genomic and Computational Biology.

Sequence data were processed with QIIME 2 v.2021.11 (5). 16S forward reads were trimmed to 220 bp and reverse reads were trimmed to 120 bp during amplicon sequence variant (ASV) calling with DADA2 (6). ITS2 paired end reads were merged with PEAR (7) without any trimming prior to processing with DADA2, as it was found to decrease the frequency of low quality bases that result in discarding of reads (6). Merged ITS2 sequences were trimmed using ITSxpress (8). Following ASV calling with DADA2, ITS2 sequences were *de novo* clustered into operational taxonomic units (OTUs) at 97% similarity using VSEARCH (9). We chose to use OTUs for fungal data and ASVs for prokaryotes because OTUs have been shown to outperform ASVs for fungi due to polymorphisms between ITS within-species copies that can result in overestimation of alpha diversity (10), while ASVs are considered the gold standard for prokaryotic 16S data (11). We refer to OTUs and ASVs collectively as “taxa” in this manuscript. ITS2 sequences and 16S sequences were assigned taxonomy using the *classify-sklearn* command in QIIME 2 (12) with the UNITE v.8.3 99% clustered all eukaryotes (13) and the SILVA version 138.2 99% (14, 15) databases, respectively. Fungal OTUs were grouped into guilds using FUNGuild (16).

**References**

1. Vilgalys R, Hester M. 1990. Rapid Genetic Identification and Mapping of Enzymatically Amplified Ribosomal DNA from Several *Cryptococcus* Species. J Bacteriol 172:4238-4246.

2. Cregger M, Veach A, Yang Z, Crouch M, Vilgalys R, Tuskan G, Schadt C. 2018. The Populus Holobiont: Dissecting the Effects of Plant Niches and Genotype on the Microbiome. Microbiome 6:1-14.

3. White TJ, Bruns T, Lee S, Taylor J. 1990. Amplification and Direct Sequencing of Fungal Ribosomal Rna Genes for Phylogenetics. PCR protocols: a guide to methods and applications 18:315-322.

4. Lane DJ, Pace B, Olsen GJ, Stahl DA, Sogin ML, Pace NR. 1985. Rapid Determination of 16s Ribosomal Rna Sequences for Phylogenetic Analyses. Proc Natl Acad Sci 82:6955-6959.

5. Bolyen E, Rideout JR, Dillon MR, Bokulich NA, Abnet CC, Al-Ghalith GA, Alexander H, Alm EJ, Arumugam M, Asnicar F. 2019. Reproducible, Interactive, Scalable and Extensible Microbiome Data Science Using Qiime 2. Nat Biotechnol 37:852-857.

6. Callahan BJ, McMurdie PJ, Rosen MJ, Han AW, Johnson AJA, Holmes SP. 2016. Dada2: High-Resolution Sample Inference from Illumina Amplicon Data. Nat Methods 13:581-583.

7. Zhang J, Kobert K, Flouri T, Stamatakis A. 2014. Pear: A Fast and Accurate Illumina Paired-End Read Merger. Bioinformatics 30:614-620.

8. Rivers AR, Weber KC, Gardner TG, Liu S, Armstrong SD. 2018. Itsxpress: Software to Rapidly Trim Internally Transcribed Spacer Sequences with Quality Scores for Marker Gene Analysis. F1000Research 7.

9. Rognes T, Flouri T, Nichols B, Quince C, Mahé F. 2016. Vsearch: A Versatile Open Source Tool for Metagenomics. PeerJ 4:e2584.

10. Tedersoo L, Bahram M, Zinger L, Nilsson RH, Kennedy PG, Yang T, Anslan S, Mikryukov V. 2022. Best Practices in Metabarcoding of Fungi: From Experimental Design to Results. Molecular ecology 31:2769-2795.

11. Callahan BJ, McMurdie PJ, Holmes SP. 2017. Exact Sequence Variants Should Replace Operational Taxonomic Units in Marker-Gene Data Analysis. The ISME journal 11:2639-2643.

12. Pedregosa F, Varoquaux G, Gramfort A, Michel V, Thirion B, Grisel O, Blondel M, Prettenhofer P, Weiss R, Dubourg V. 2011. Scikit-Learn: Machine Learning in Python. the Journal of machine Learning research 12:2825-2830.

13. Nilsson RH, Larsson K-H, Taylor AFS, Bengtsson-Palme J, Jeppesen TS, Schigel D, Kennedy P, Picard K, Glöckner FO, Tedersoo L. 2019. The Unite Database for Molecular Identification of Fungi: Handling Dark Taxa and Parallel Taxonomic Classifications. Nucleic Acids Res 47:D259-D264.

14. Quast C, Pruesse E, Yilmaz P, Gerken J, Schweer T, Yarza P, Peplies J, Glöckner FO. 2012. The Silva Ribosomal Rna Gene Database Project: Improved Data Processing and Web-Based Tools. Nucleic Acids Res 41:D590-D596.

15. Yilmaz P, Parfrey LW, Yarza P, Gerken J, Pruesse E, Quast C, Schweer T, Peplies J, Ludwig W, Glöckner FO. 2014. The Silva and “All-Species Living Tree Project (Ltp)” Taxonomic Frameworks. Nucleic Acids Res 42:D643-D648.

16. Nguyen NH, Song Z, Bates ST, Branco S, Tedersoo L, Menke J, Schilling JS, Kennedy PG. 2016. Funguild: An Open Annotation Tool for Parsing Fungal Community Datasets by Ecological Guild. Fungal Ecol 20:241-248.
