# Supplementary material for: Time-series RNA metabarcoding of the active Populus tremuloides root microbiome reveals hidden temporal dynamics and dormant core members
Source: mSystems. 2025 Nov 7;10(12):e00285-25. doi: 10.1128/msystems.00285-25 (PMC12710371; doi:10.1128/msystems.00285-25)

**Supplemental Figure S1.** Mean understory vegetation cover at each site determined with a line point intercept method averaged across the five plots at each site.

**
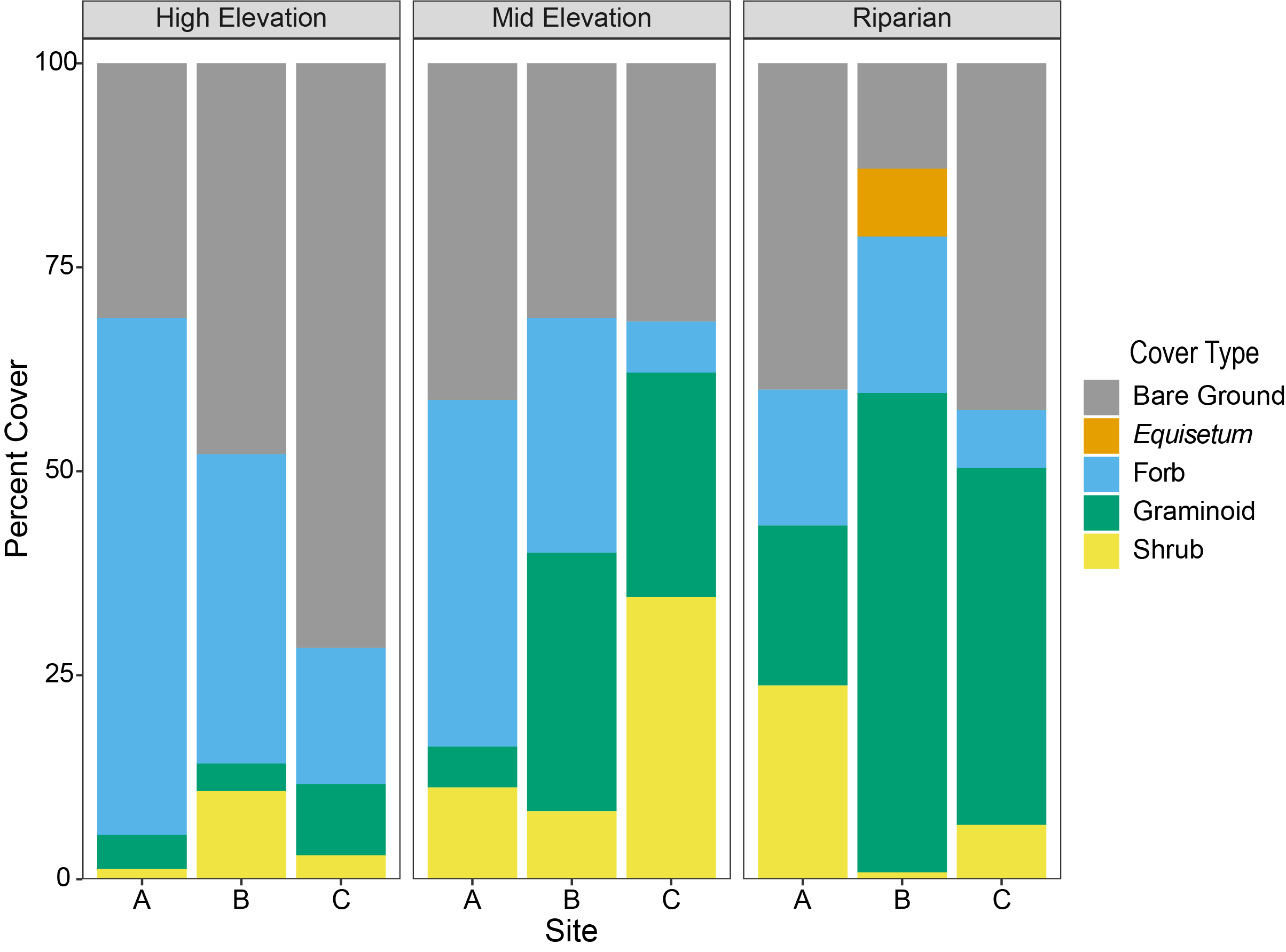
**

**Supplemental Figure S2.** Soil properties of soils collected at a single timepoint in May 2021. Pairwise significance between site types determined by mixed models is indicated by letters. Plots without letters had no significant pairwise tests.


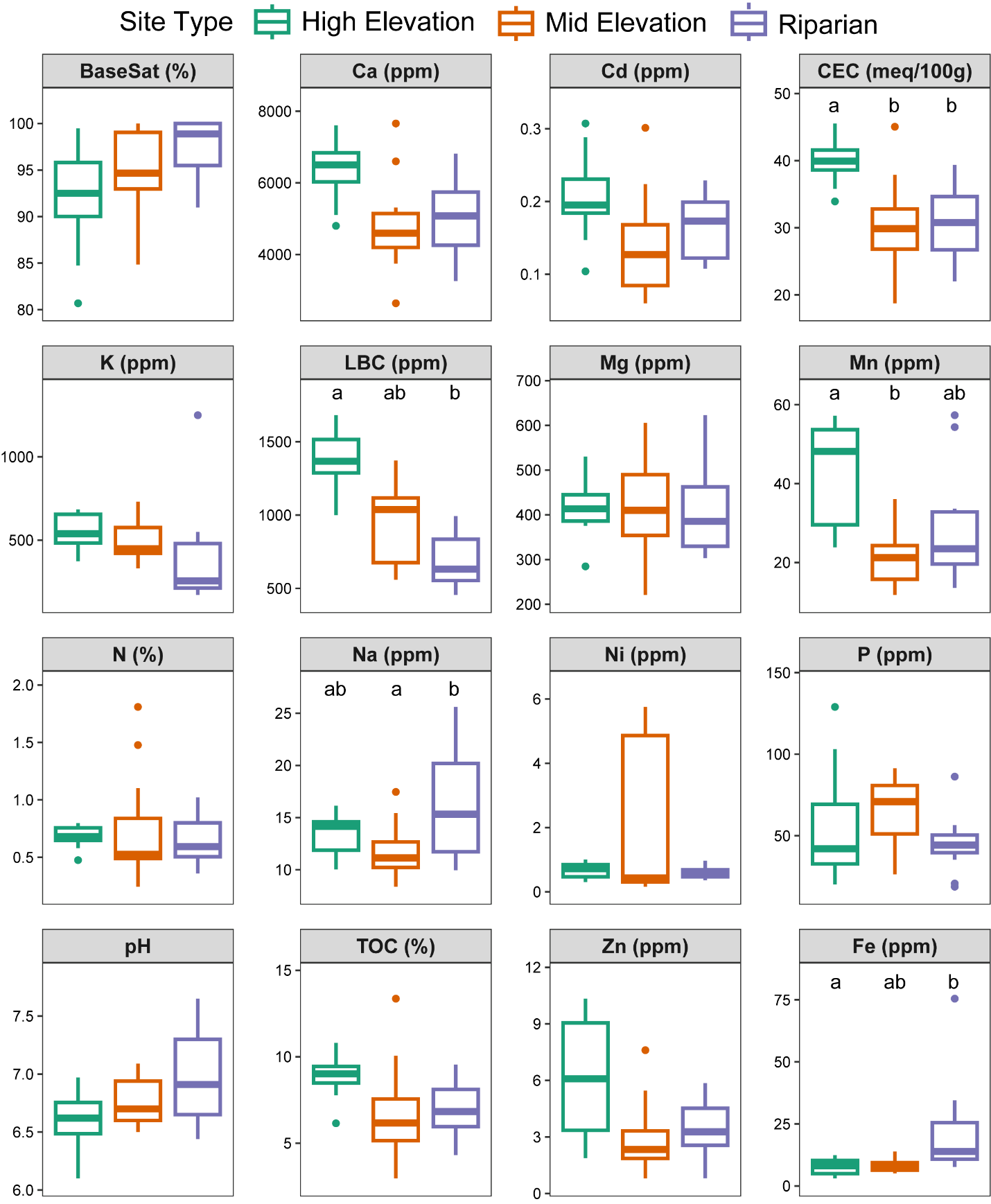


**Supplemental Figure S3.** Average soil moisture and temperature measurements over the course of the growing season (June-October) taken at each plot. Each box represents a different site.


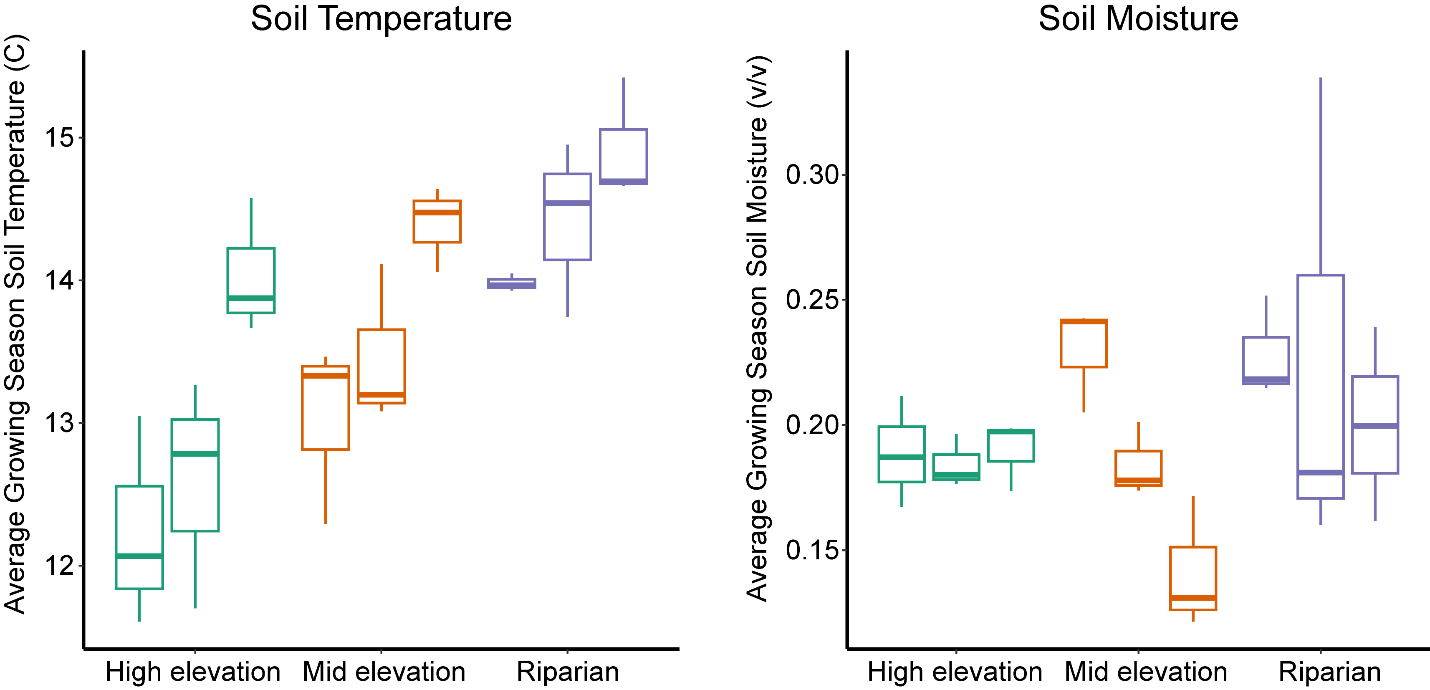


**Supplemental Figure S4.** Ion fluxes determined by plant root simulators across the site types and seasons. Significance of these differences is displayed in Table S1. Fluxes of NH_4_, Cu, Pb, and Cd were also measured, but were generally below the limit of detection and were not analyzed further.

**
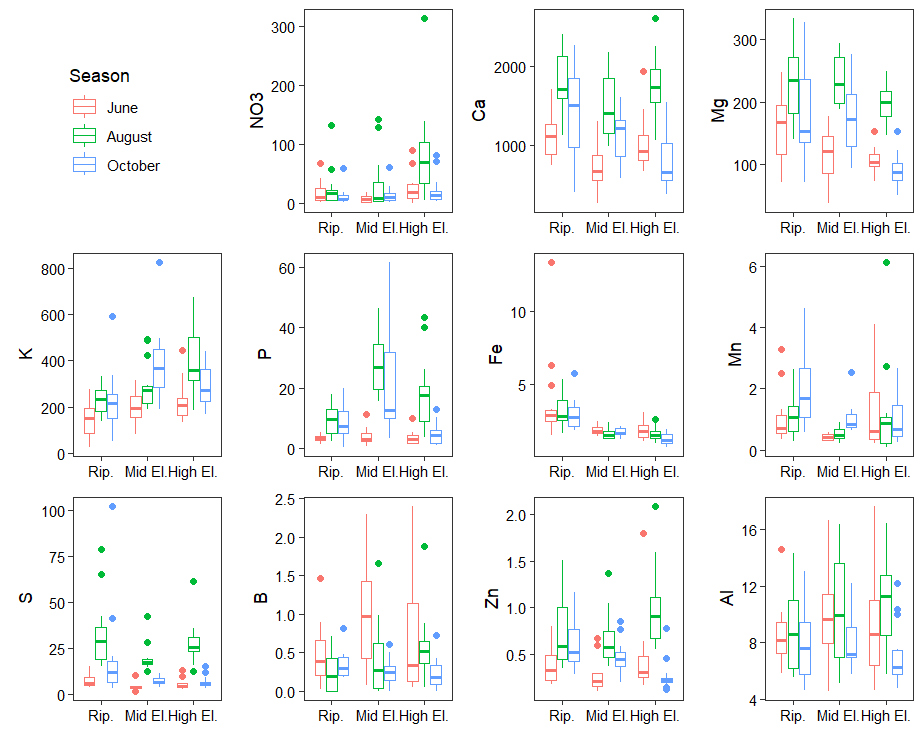
**

**Supplemental Figure S5.** Mean within-site beta dispersion for the ITS and 16S datasets calculated with Bray-Curtis dissimilarity. Beta dispersion was calculated as the distance to centroid for each unique combination site, season, and community type (total vs. active). Error bars represent the standard error.


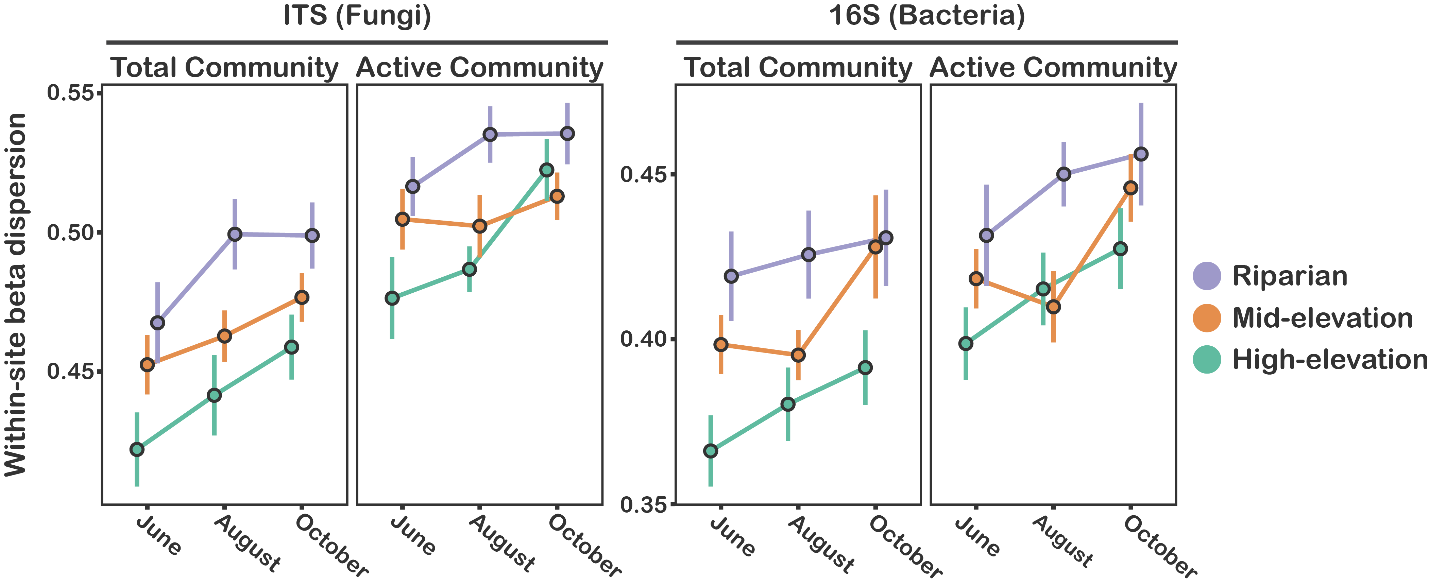


**Supplemental Figure S6.** Distance decay relationships of community composition across seasons for active and total communities of fungi and bacteria. Distances between plots in the same site are shown in red and distances between plots in different sites are shown in blue. P values and r values from Mantel tests are displayed for each plot.


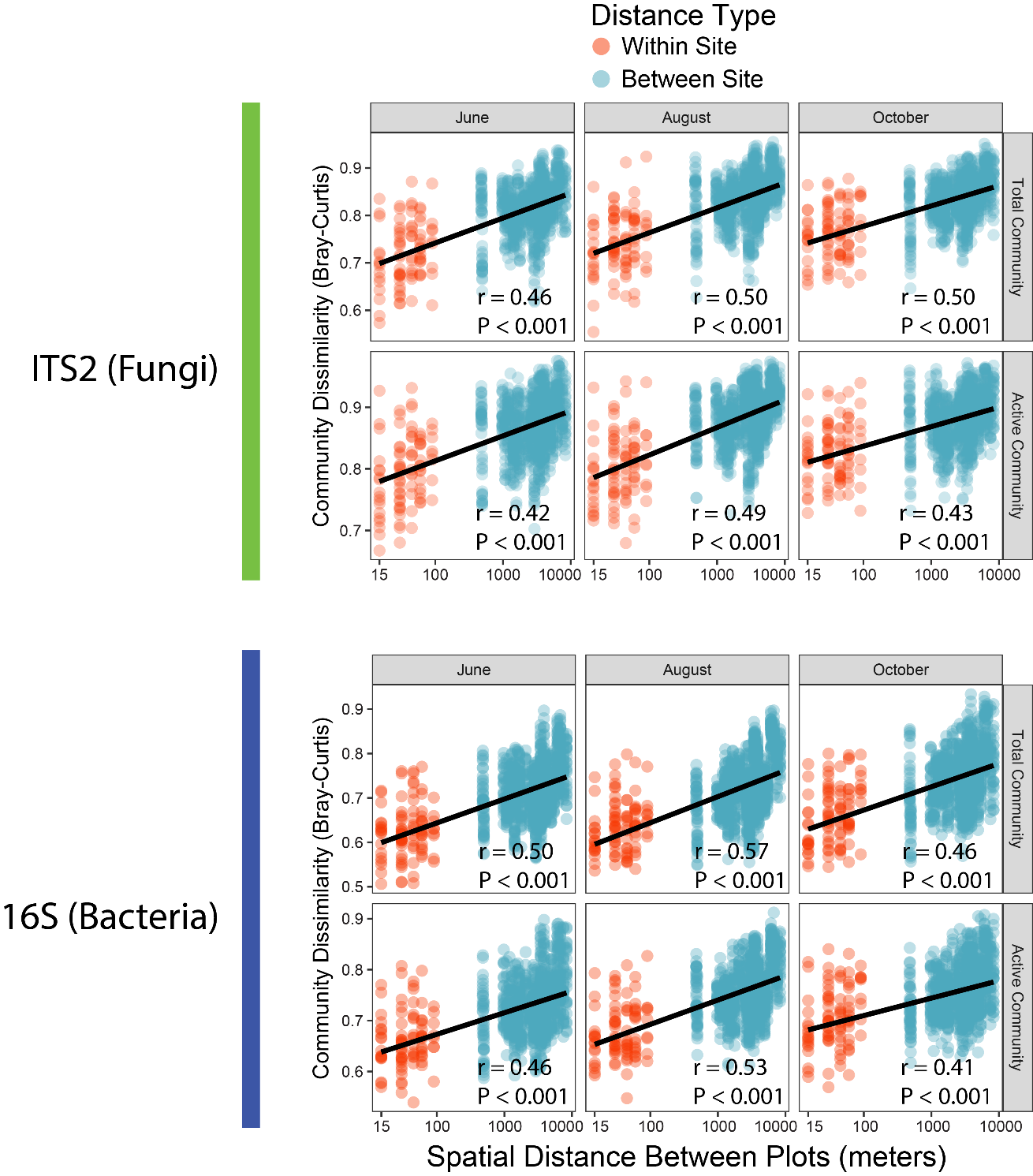


**Supplemental Figure S7.** Relationship between NO_3_ flux and the relative sequence abundance of the archaeal phylum Thermoproteota in the total and active community determined by 16S sequencing.

**
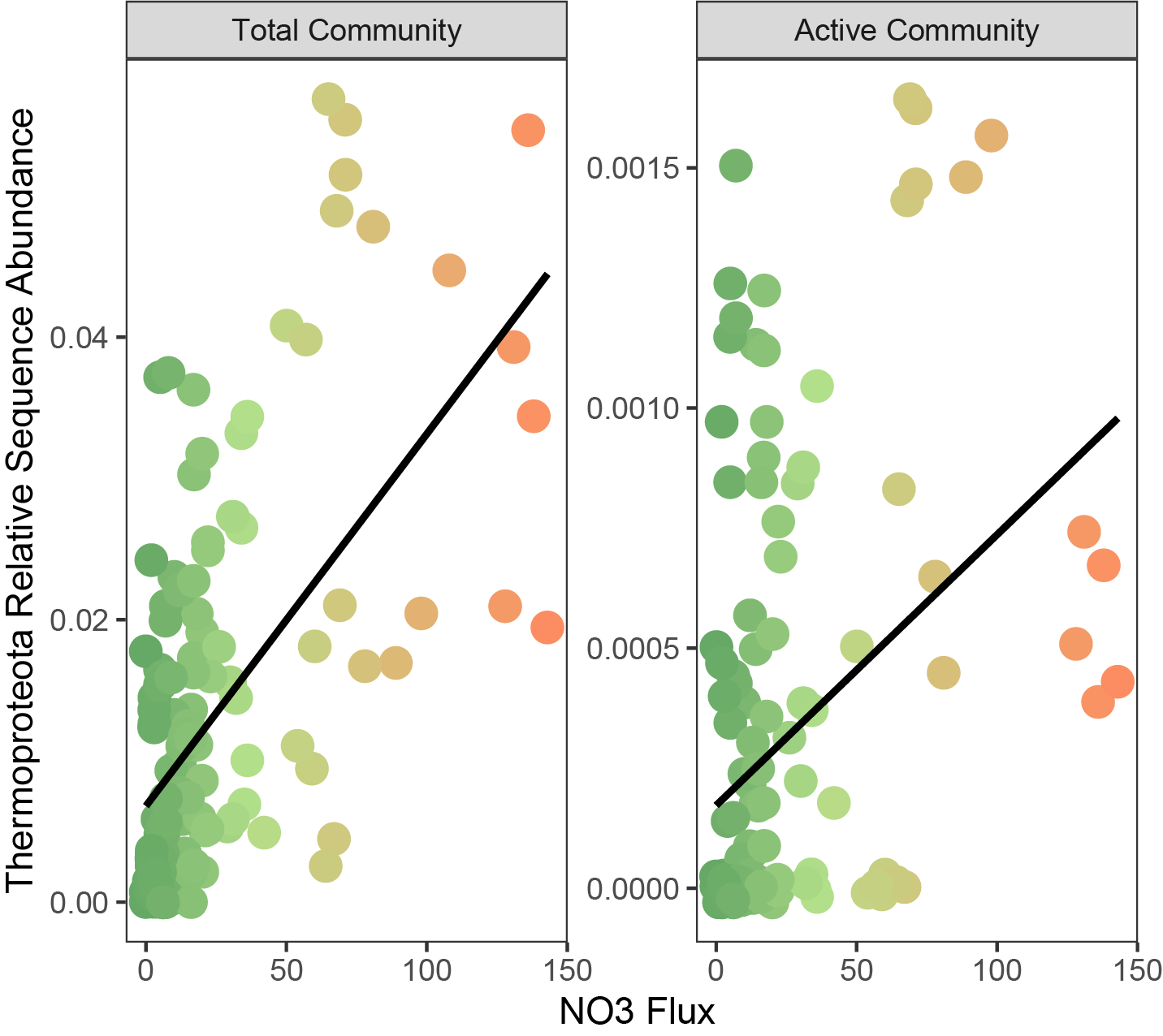
**

**Supplemental Figure S8.** Occupancy of all fungal and bacterial taxa included in the total and active core communities. Occupancy of 1 indicates presence in all samples, and occupancy of 0 indicates presence in no samples


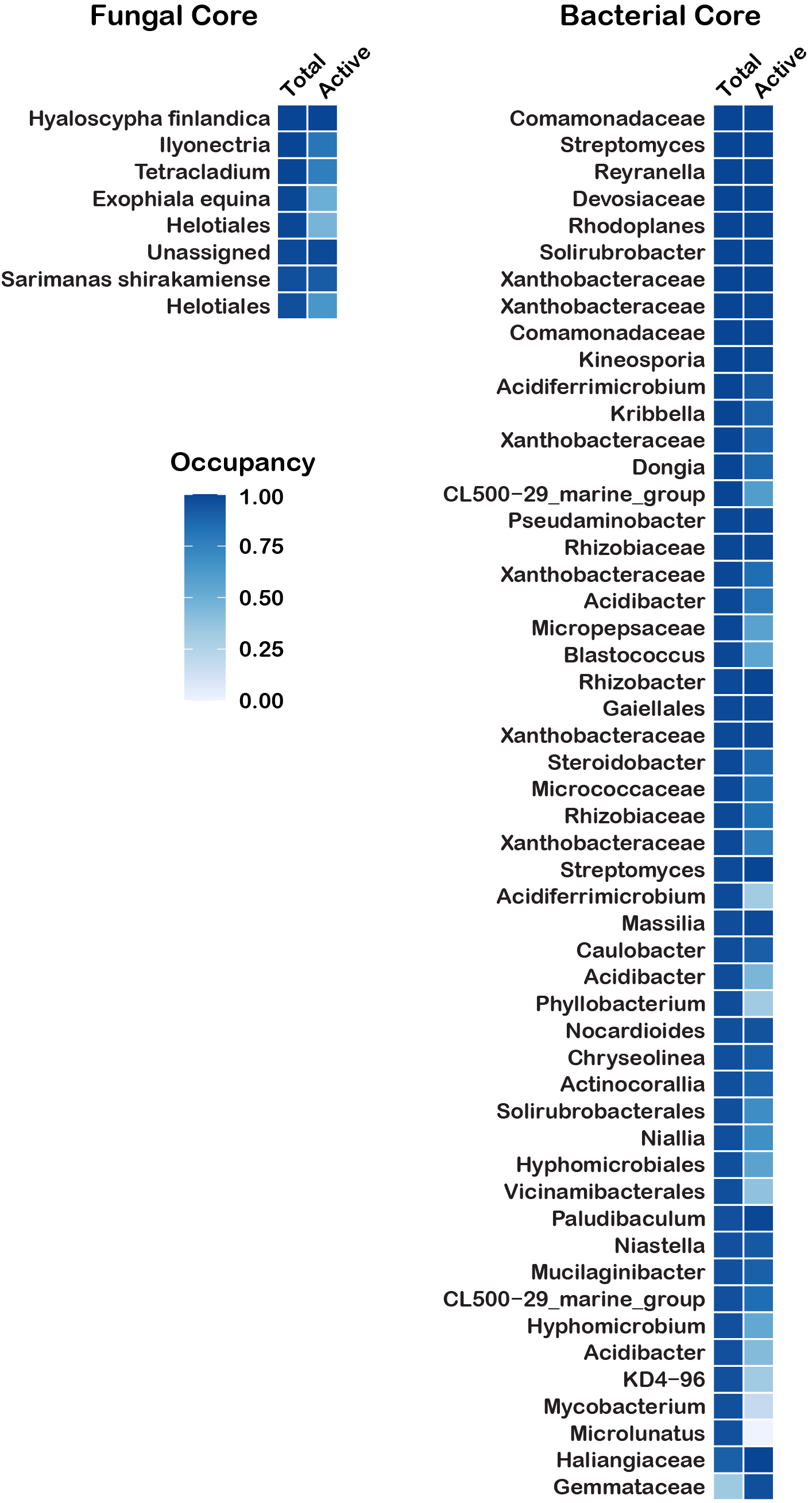

Supplement: File S2 — Figures S1 to S8. [file msystems.00285-25-s0002.docx]
