# Supplementary material for: Time-series RNA metabarcoding of the active Populus tremuloides root microbiome reveals hidden temporal dynamics and dormant core members
Source: mSystems. 2025 Nov 7;10(12):e00285-25. doi: 10.1128/msystems.00285-25 (PMC12710371; doi:10.1128/msystems.00285-25)
Supplement: File S3 — Tables S1 to S11. [file msystems.00285-25-s0003.docx]

**Supplemental Table S1.** P-values of linear mixed models testing for the effects of site type and season on ion fluxes measured with Plant Root Simulators. Significance is determined by a P < 0.05 and is highlighted in green

| Response | SiteType_p_value | Season_p_value | Interaction_p_value |
| --- | --- | --- | --- |
| NO3 | 0.19403104 | 1.75E-06 | 0.007261109 |
| Ca | 0.389180676 | 3.10E-35 | 2.74E-05 |
| Mg | 0.207338783 | 1.17E-41 | 0.000151054 |
| K | 0.00244165 | 1.94E-08 | 0.002502492 |
| P | 0.036627124 | 4.36E-24 | 6.51E-08 |
| Fe | 1.07E-05 | 0.058754552 | 0.707657484 |
| Mn | 0.457655027 | 0.004357443 | 0.006196071 |
| S | 0.164432686 | 3.20E-21 | 0.076473991 |
| B | 0.07636019 | 1.59E-06 | 0.019869014 |
| Zn | 0.288711842 | 6.49E-14 | 0.000174301 |
| Al | 0.309418868 | 0.000255782 | 0.35814302 |

**Supplemental Table S2**. ANOVA table of linear mixed effects model testing for effects of site type, season, and metabarcoding method (DNA vs RNA) on fungal ITS2 Shannon diversity

| ITS2 (fungal) Shannon Diversity | | | |
| --- | --- | --- | --- |
|  | Chisq | Df | *P* |
| SiteType | 18.5648 | 2 | 9.31E-05 |
| Season | 19.5858 | 2 | 5.59E-05 |
| DNA_RNA | 15.8653 | 1 | 6.80E-05 |
| SiteType:Season | 5.3466 | 4 | 0.2536 |
| SiteType:DNA_RNA | 2.84 | 2 | 0.2417 |
| Season:DNA_RNA | 0.3388 | 2 | 0.8442 |
| SiteType:Season:DNA_RNA | 0.9334 | 4 | 0.9197 |

**Supplemental Table S3**. ANOVA table of linear mixed effects model testing for effects of site type, season, and metabarcoding method (DNA vs RNA) on prokaryotic 16S Shannon diversity

| 16S (prokaryotic) Shannon Diversity | | | |
| --- | --- | --- | --- |
|  | Chisq | Df | *P* |
| SiteType | 14.855 | 2 | 5.95E-04 |
| Season | 11.6267 | 2 | 0.0029874 |
| DNA_RNA | 710.0664 | 1 | < 2.2e-16 |
| SiteType:Season | 4.7856 | 4 | 0.3100168 |
| SiteType:DNA_RNA | 2.9685 | 2 | 0.2266683 |
| Season:DNA_RNA | 0.941 | 2 | 0.6246959 |
| SiteType:Season:DNA_RNA | 6.7062 | 4 | 0.1522505 |

**Supplemental Table S4.** PERMANOVA table testing for effects of site type, season, and metabarcoding method (DNA vs RNA) on fungal ITS2 community composition using Bray-Curtis dissimilarity with the full dataset. The model uses sequential sum of squares and terms were entered in the order listed below.

| ITS2 (Fungal) PERMANOVA - Full Dataset | | | | | |
| --- | --- | --- | --- | --- | --- |
|  | Df | SumOfSqs | R^2^ | F | *P* |
| SiteType | 2 | 6.782 | 0.06686 | 13.2406 | 0.001 |
| Site | 6 | 9.047 | 0.08918 | 5.8871 | 0.001 |
| Plot | 36 | 22.235 | 0.21918 | 2.4116 | 0.001 |
| Season | 2 | 1.529 | 0.01507 | 2.9854 | 0.001 |
| DNA_RNA | 1 | 5.764 | 0.05682 | 22.5053 | 0.001 |

**Supplemental Table S5.** PERMANOVA table testing for effects of site type, season, and metabarcoding method (DNA vs RNA) on prokaryotic 16S community composition using Bray-Curtis dissimilarity with the full dataset. The model uses sequential sum of squares and terms were entered in the order listed below.

| 16S (Prokaryotic) PERMANOVA - Full Datatset | | | | | |
| --- | --- | --- | --- | --- | --- |
|  | Df | SumOfSqs | R^2^ | F | *P* |
| SiteType | 2 | 5.011 | 0.06138 | 11.9182 | 0.001 |
| Site | 6 | 6.981 | 0.0855 | 5.5341 | 0.001 |
| Plot | 36 | 12.617 | 0.15454 | 1.6671 | 0.001 |
| Season | 2 | 1.053 | 0.0129 | 2.5045 | 0.001 |
| DNA_RNA | 1 | 9.733 | 0.11921 | 46.2949 | 0.001 |

**Supplemental Table S6.** PERMANOVA table testing for effects of site type and season on fungal ITS2 community composition using Bray-Curtis dissimilarity with the DNA-only data subset. The model uses sequential sum of squares and terms were entered in the order listed below.

| ITS2 (Fungal) PERMANOVA - DNA Dataset only | | | | | |
| --- | --- | --- | --- | --- | --- |
|  | Df | SumOfSqs | R^2^ | F | *P* |
| SiteType | 2 | 4.724 | 0.10564 | 9.9422 | 0.001 |
| Site | 6 | 5.651 | 0.12637 | 3.9645 | 0.001 |
| Plot | 36 | 13.19 | 0.29494 | 1.5421 | 0.001 |
| Season | 2 | 0.723 | 0.01616 | 1.5205 | 0.001 |

**Supplemental Table S7.** PERMANOVA table testing for effects of site type and season on fungal ITS2 community composition using Bray-Curtis dissimilarity with the RNA-only data subset. The model uses sequential sum of squares and terms were entered in the order listed below.

| ITS2 (Fungal) PERMANOVA - RNA Dataset only | | | | | |
| --- | --- | --- | --- | --- | --- |
|  | Df | SumOfSqs | R^2^ | F | *P* |
| SiteType | 2 | 3.729 | 0.07257 | 6.1412 | 0.001 |
| Site | 6 | 5.411 | 0.10531 | 2.9707 | 0.001 |
| Plot | 36 | 14.5 | 0.2822 | 1.3268 | 0.001 |
| Season | 2 | 1.332 | 0.02592 | 2.194 | 0.001 |

**Supplemental Table S8.** PERMANOVA table testing for effects of site type and season on prokaryotic 16S community composition using Bray-Curtis dissimilarity with the DNA-only data subset. The model uses sequential sum of squares and terms were entered in the order listed below.

| 16S (Prokaryotic) PERMANOVA - DNA Dataset only | | | | | |
| --- | --- | --- | --- | --- | --- |
|  | Df | SumOfSqs | R^2^ | F | *P* |
| SiteType | 2 | 3.831 | 0.10886 | 9.91 | 0.001 |
| Site | 6 | 4.841 | 0.13756 | 4.1742 | 0.001 |
| Plot | 36 | 8.888 | 0.25255 | 1.2772 | 0.001 |
| Season | 2 | 0.622 | 0.01768 | 1.6092 | 0.003 |

**Supplemental Table S9.** PERMANOVA table testing for effects of site type and season on prokaryotic 16S community composition using Bray-Curtis dissimilarity with the RNA-only data subset. The model uses sequential sum of squares and terms were entered in the order listed below.

| 16S (Prokaryotic) PERMANOVA - RNA Dataset only | | | | | |
| --- | --- | --- | --- | --- | --- |
|  | Df | SumOfSqs | R^2^ | F | *P* |
| SiteType | 2 | 2.761 | 0.07464 | 6.246 | 0.001 |
| Site | 6 | 4.574 | 0.12364 | 3.449 | 0.001 |
| Plot | 36 | 9.755 | 0.26371 | 1.226 | 0.001 |
| Season | 2 | 0.895 | 0.02418 | 2.0239 | 0.001 |

**Supplemental Table S10.** ANOVA table of linear mixed effects model testing for effects of site type, season, and metabarcoding method (DNA vs RNA) on beta dispersion on the ITS2 Bray-Curtis dissimilarity matrix

| ITS2 Beta Dispersion of Bray-Curtis Dissimilarity | | | |
| --- | --- | --- | --- |
|  | Chisq | Df | *P* |
| SiteType | 11.812 | 2 | 0.002723 |
| Season | 31.0868 | 2 | 1.78E-07 |
| DNA_RNA | 131.496 | 1 | < 2.20E-16 |
| SiteType:Season | 7.6189 | 4 | 0.10658 |
| SiteType:DNA_RNA | 2.3646 | 2 | 0.306574 |
| Season:DNA_RNA | 1.9484 | 2 | 0.377496 |
| SiteType:Season:DNA_RNA | 1.4382 | 4 | 0.837528 |

**Supplemental Table S11.** ANOVA table of linear mixed effects model testing for effects of site type, season, and metabarcoding method (DNA vs RNA) on beta dispersion on the 16S Bray-Curtis dissimilarity matrix

| 16S Beta Dispersion of Bray-Curtis Dissimilarity | | | |
| --- | --- | --- | --- |
|  | Chisq | Df | *P* |
| SiteType | 12.3842 | 2 | 0.002046 |
| Time | 19.8268 | 2 | 4.95E-05 |
| DNA_RNA | 26.6897 | 1 | 2.39E-07 |
| SiteType:Time | 5.3543 | 4 | 0.252833 |
| SiteType:DNA_RNA | 2.4436 | 2 | 0.294705 |
| Time:DNA_RNA | 0.1952 | 2 | 0.907008 |
| SiteType:Time:DNA_RNA | 0.5132 | 4 | 0.972201 |
